# Tonicity inversely modulates lipocalin-2 (Lcn2/24p3/NGAL) receptor (SLC22A17) and Lcn2 expression via Wnt/β-catenin signaling in renal inner medullary collecting duct cells: implications for cell fate and bacterial infection

**DOI:** 10.1186/s12964-018-0285-3

**Published:** 2018-11-07

**Authors:** R. Betten, B. Scharner, S. Probst, B. Edemir, N. A. Wolff, C. Langelueddecke, W.-K. Lee, F. Thévenod

**Affiliations:** 1 Department of Physiology, Pathophysiology & Toxicology and ZBAF (Centre for Biomedical Education and Research), Faculty of Health, School of Medicine, Witten/Herdecke University, Stockumer Str 12 (Thyssenhaus), D-58453 Witten, Germany; 2 Department of Medicine, Hematology and Oncology, Martin Luther University Halle-Wittenberg, 06120 Halle (Saale), Germany

**Keywords:** Kidney, hypertonicity, osmotic stress, cell death, proliferation, lipocalin-2, lipocalin-2 receptor, Wnt/beta-catenin, lipopolysaccharide

## Abstract

**Background:**

We have previously evidenced apical expression of the 24p3/NGAL/lipocalin-2 receptor (Lcn2-R; *SLC22A17*) in inner medullary collecting duct (IMCD) cells, which are present *in vivo* in a hyperosmotic/-tonic environment that activates canonical Wnt/β-catenin signaling. The localization of Lcn2-R in the inner medulla is intriguing considering local bacterial infections trigger toll-like receptor-4 (TLR-4)-mediated secretion of the bacteriostatic Fe^3+^-free (apo-)Lcn2.

**Aim:**

To determine the effects of osmolarity/tonicity changes, Wnt/β-catenin and TLR-4 activation on Lcn2-R and Lcn2 expression and cell viability in rat primary IMCD and mouse (m)IMCD3 cells.

**Methods:**

Normosmolarity/-tonicity was 300 mosmol/l whereas hyperosmolarity/-tonicity was induced by adding 100 mmol/l NaCl + 100 mmol/l urea (600 mosmol/l, 1-7 days). Lcn2-R and Lcn2 expression were determined by qPCR, immunoblotting, flow cytometry and immunofluorescence microscopy. β-catenin was silenced by RNAi. Cell viability/death was determined with MTT and LDH release assays. TLR-4 was activated by bacterial lipopolysaccharides (LPS).

**Results:**

Hyperosmotic/-tonic media upregulated Lcn2-R by ~4-fold and decreased Lcn2 expression/secretion, along with Wnt/β-catenin activation, in IMCD cells. These effects of hyperosmotic/-tonic media on Lcn2-R/Lcn2 expression were reverted by normosmolarity/-tonicity, β-catenin silencing and/or LPS. Exposure of cells with endogenous or stably overexpressing Lcn2-R to apo-Lcn2 or LPS decreased cell viability.

**Conclusions:**

Lcn2-R upregulation and Lcn2 downregulation via Wnt/β-catenin may promote adaptive osmotolerant survival of IMCD cells in response to hyperosmolarity/-tonicity whereas Lcn2 upregulation and Lcn2-R downregulation via TLR-4 and/or normosmolarity/-tonicity may protect IMCD cells against bacterial infections and prevent autocrine death induction by Lcn2.

**Electronic supplementary material:**

The online version of this article (10.1186/s12964-018-0285-3) contains supplementary material, which is available to authorized users.

## Background

The cells of the inner medullary collecting duct (IMCD) are key elements of the nephron in the process of urinary concentration and dilution [1]. For these processes to be operative, interstitial hyperosmolarity/-tonicity needs to be established by accumulation of high interstitial levels of NaCl and urea [2, 3]. However, the hypertonic effect of high NaCl is stressful to cells, may alter cellular functions and even kill cells by apoptosis (reviewed in [4]). Cells can counteract high osmolality stress by initiating survival mechanisms that activate the transcription factor TonEBP/OREBP/NFAT5 (reviewed in [4]). These survival mechanisms include accumulation of organic osmolytes and increased expression of heat shock proteins through numerous pathways, resulting in osmotolerance. Hence, the unique hypertonic environment in the renal medulla induces a nephron segment-specific gene expression pattern [5].

Neutrophil gelatinase-associated lipocalin (NGAL in humans, siderocalin/24p3 in rodents) or lipocalin-2 (Lcn2) is secreted by cells [6] in an Fe^3+^-free apo form, which binds Fe^3+^ through association with bacterial siderophores (forming holo-Lcn2). Apo-Lcn2 hence plays an important role in antibacterial innate immunity [7]. Several studies have demonstrated that apo-Lcn2 induces apoptosis in Lcn2-R expressing cells [8, 9], whereas holo-Lcn2 is anti-apoptotic and may even promote cell proliferation, e.g. in cancer cells [10]. Lcn2 is expressed in cells as a non-glycosylated ~22-kDa precursor and a glycosylated ~25-kDa mature Lcn2, but only the latter is secreted [6, 11]. Lcn2 is also secreted by various epithelia, including the kidney; however its function there is less clear. It has been proposed that holo-Lcn2 stimulates epithelial growth and differentiation, and promotes repair and regeneration of damaged epithelia, e.g. during acute kidney injury [12], (reviewed in [13]). A recent study has implicated Lcn2 secretion from collecting duct (CD) α-intercalated cells as a bacteriostatic defense mechanism against pathogenic bacteria in the urine [14]. Secretion was induced by the bacterial wall component lipopolysaccharide (LPS) within the renal collecting duct (CD) via Toll-like cell surface receptor-4 (TLR-4) activation [14], likely involving NF-κB activation [15].

A receptor for Lcn2 (Lcn2-R/*Slc22a17*/24p3-R) has been cloned (MM ~60kDa) [8] and is expressed in multiple epithelial tissues, including the renal nephron. In the rodent kidney, Lcn2-R was detected apically in distal convoluted tubules and CD [16]. The most abundant localization of Lcn2-R in the kidney is the medulla [16] thus strongly implying a relationship with the hyperosmolar environment. Experimental evidence in cultured cells and *in vivo* [16, 17] indicates that Lcn2-R is a high-affinity protein receptor in the distal nephron. The data suggest that physiologically it is responsible for exhaustive protein reabsorption to clear the final urine from proteins, or to limit losses associated with proteinuric renal diseases [18]. In fact, Lcn2-R affinity for Lcn2 is ~1000x higher (*K*_*D*_ ~90pM) [19] than that of megalin (*K*_*D*_ ~60nM) [20], the high-capacity receptor for endocytic reabsorption of filtered proteins in the proximal tubule [21].

Little is known about the physiological regulation of Lcn2-R and Lcn2 expression, which may be interlinked. Inverse co-regulation of Lcn2 and Lcn2-R was observed by Green and coworkers [8, 9] who showed in murine leukemia cell models that the oncogene BCR-ABL increases Lcn2 and represses Lcn2-R expression. Lcn2 and Lcn2-R are also co-regulated by the Wnt/β-catenin pathway, which is involved in survival, growth and proliferation [22] and can be activated by hyperosmotic stress [23, 24]. In murine mammary epithelial C57MG cells, Ziegler et al. [25, 26] demonstrated that overexpression of Wnt-1 decreases *Lcn2*. Furthermore, Wnt-1 activity affected splicing of Lcn2-R transcripts, indicating co-regulation of *Lcn2* and Lcn2-R expression [25].

The aim of the study was to determine the role of osmolarity/tonicity and Wnt/β-catenin signaling on Lcn2 and Lcn2-R expression in rat primary IMCD and mouse (m)IMCD3 cells exposed to norm- and hyperosmotic/-tonic media. The data indicate that Lcn2-R upregulation and Lcn2 downregulation in hyperosmotic/-tonic media is mediated by activation of Wnt/β-catenin signaling and protects IMCD cells against Lcn2-induced damage and death. In contrast, LPS upregulates Lcn2 but downregulates Lcn2-R in IMCD cells, which may also be cytoprotective in the context of urinary tract infections (UTIs). The significance of the inverse regulation of Lcn2-R and Lcn2 as a protective measure in the context of normosmotic conditions and/or bacterial infection is discussed.

## Methods

### Materials

Recombinant Fe^3+^-free rat apo-Lcn2 was from Enzo Life Sciences (ALX-201-417-C050). Lipopolysaccharides from Escherichia coli (E coli) (cat. # L3129) were from Sigma-Aldrich. All other reagents were purchased at the highest purity grade possible. Materials were dissolved either in water, ethanol, or dimethyl sulfoxide (DMSO). In control experiments, solvents were added to cells at concentrations not exceeding 0.2%. Antibodies are listed in Table 1.

**Table 1:** Primary antibodies

Immunogen	Host species	Manufacturer	Catalog #	Application	Dilution
Lcn2 (rat)	goat	RD Systems	AF3508	IB	1:200-300
Lcn2 (mouse)	rabbit	Abcam	Ab63929	IF	1:250-500
Lcn2-R (C-term) (rat)	rabbit	Immunoglobe	Ig-1086	IB	1:250-2,000
Lcn2-R (N-term) (rat)	rabbit	Immunoglobe	Ig-1095	IF	1:100-1000
β-actin	mouse	Sigma-Aldrich	A5316	IB	1:20,000
β-catenin (mouse)	mouse	BD Biosciences	610153	IB	1:2,000-5,000
GSK-3β (rat)	mouse	BD Biosciences	610201	IB	1:500
Phospho-GSK-3 β (Ser9) (human)	rabbit	Cell Signaling Technology	9336	IB	1:500
Na^+^,K^+^-ATPase α1-subunit (human)	rabbit	Cell Signaling Technology	3010S	IB IF	1:250-500 1:100
FLAG-M2	mouse	Sigma-Aldrich	F3165	IB	10μg/ml

*IB* = immunoblotting. *IF* = immunofluorescence

### Culture of mIMCD3 cells and transient transfections

The mouse mIMCD3 cell line [27] was obtained from ATCC (cat. # ATCC CRL-2123). Cells (passage numbers 20-30) were cultured in Dulbecco’s modified Eagle’s medium (DMEM)/F-12 (1:1) (Gibco cat. # 31330) supplemented with 10% fetal bovine serum (FBS), 50 U/ml penicillin, and 50 μg/ml streptomycin. Cells were cultured in 25 cm^2^ standard tissue culture flasks (Sarstedt) at 37^o^C in a humidified incubator with 5% CO_2_ and were passaged twice a week upon reaching 90% confluency.

For transient transfections, 1.0-1.3 x 10^5^ mIMCD3 cells were seeded in 6-well plates. After 24h, cells were ~40% confluent and transfected with 25 nM β-catenin (Gene name: *Ctnnb1*) SMART-pool siRNA (Dharmacon cat. # L-040628-00-0005) or siRNA duplex negative control (Eurogentec cat. # SR-CL000-005) in standard culture medium without antibiotics using Lipofectamine RNAiMAX (Thermo Fisher Scientific) according to manufacturer’s instructions. After 6 h, medium was exchanged to 300 and 600 mosmol/l media with or without FBS and incubated for up to 48 h.

### Isolation and culture of rat primary IMCD cells

Inner medullary collecting duct (IMCD) cells were prepared from inner medullas including papilla of sacrificed Wistar rats (license # 84-02.05.20.11.256), as previously described [28]. Briefly, the inner medullas including papilla of sacrificed Wistar rats were removed, cut into small pieces, and digested in phosphate-buffered saline (PBS) containing 0.2% hyaluronidase (Sigma) and 0.2% collagenase type CLS-II (Sigma) at 37°C for 90 min. Cells were centrifuged at 450 x *g*, resuspended in PBS and centrifuged once more. Cells were seeded on glass coverslips coated with collagen type IV (Becton-Dickinson) at a density of 10^5^ cells/cm^2^ and cultured in DMEM containing 100 IU/ml penicillin, 100 μg/ml streptomycin, 0.2% glutamine, 1% nonessential amino acids and 1% ultroser G (BioSepra Inc.) in an 8% CO_2_ atmosphere.

### Osmolarity experiments

Unless otherwise indicated, in osmolarity experiments, cell lines were cultured for 24 h in standard culture medium after plating before osmolarity was adjusted to 600 mosmol/l by the addition of 100 mmol/l NaCl and 100 mmol/l urea from 3 M and 6 M stock solutions to normosmotic/-tonic culture media (300 mosmol/l), respectively, and cultured in normo-/hyperosmotic medium for up to 72 h (for simplification the term “osmolarity” henceforth refers to osmolarity and tonicity). Proliferation was reduced in mIMCD3 cells exposed to 600 mosmol/l media (Additional file 1: Figure S1A) along with initial decrease of cell cycle and proliferation genes *Myc* and *Ccnd1* mRNA, which returned to normal (*Ccnd1*) or was even elevated (*Myc*) at 72 h (Additional file 1: Figure S1B). This confirms that a hyperosmotic environment induces cell stress with cell cycle arrest and inhibition of proliferation [4]. Reactive adaptation to hyperosmolarity was supported by up-regulation of Na^+^/K^+^-ATPase at 72 h (Additional file 1: Figure S1C, bottom; Additional file 1: Figure S1D, lanes 2 and 4), which is known to contribute to osmoprotection of primary IMCD [29] and mIMCD3 cells [30].

For primary cells, osmolarity was adjusted to 600 mosmol/l by addition of 100 mmol/l NaCl and 100 mmol/l urea to normosmotic culture media in order to preferentially select for IMCD cells. After 24 h, the medium was replaced by normosmotic or hyperosmotic media by adding the appropriate concentration of osmolytes (no additions for 300 mosmol/l and or mmol/l NaCl and 100 mmol/l urea for 600 mosmol/l) and experiments were performed one week later.

### Plasmid construction and generation of CHO-K1 cells stably expressing LCN2-R

The plasmid encoding C-terminally FLAG-tagged full-length human (h)LCN2-R isoform A (GenBank accession number NM_020372.3) was generated by replacing the sequence encoding rLcn2R with hLCN2-R isoform A by restriction digestion and ligation (Ligate-IT, VWR/Affymetrix) in the plasmid previously described [31] (kindly provided by Dr. N. Coudevylle, Max F. Perutz Laboratories, University of Vienna, Austria). The sequence was then converted to hLCN2-R isoform B (GenBank accession number NM_016609.4) (which – in preliminary experiments – appeared to be more efficiently expressed in the plasma membrane; N.A.W. & F.T., *unpublished observations*) by site-directed mutagenesis (QuikChange II Site-Directed Mutagenesis Kit; Agilent Technologies) as per manufacturer’s instructions, using the following primers: forward 5’-CTGGGCCTGTGGGATTATCTGAACGAGGCTGCC-3′, reverse 5’-GGCAGCCTCGTTCAGATAATCCCACAGGCCCAG-3’, 18 cycles of 30 s at 95°C, 60 s at 55°C, and 8 min at 68°C. All constructs were verified by sequencing.

To generate stable CHO-K1 clones, cells seeded at 2.5 x 10^4^ cells/cm^2^ were transfected 24 h later with hLCN2-R-FLAG isoform B construct, or empty pcDNA3.1 vector using Lipofectamine LTX Plus (Thermo Fisher) according to manufacturer’s instructions, selected for >8 weeks under 600 μg/ml G418 with medium change every 2-3 days, and passaged upon reaching confluency. Stable clones were further selected with G418 by two rounds of serial dilution and plating at dilutions corresponding to a density of about 0.5, 3 and 10 cells / well in 96-well plates. Wells containing individual colonies were controlled by microscopy and hLCN2-R-FLAG isoform B expression was verified by reverse transcriptase (RT) PCR (Additional file 2: Figure S2A) and immunoblotting (Additional file 2: Figure S2B).

### Cell viability assay of mIMCD3 and CHO-K1 cells

1.5 x 10^4^ mIMCD3 cells were seeded per well in 48-well plates and cultured for 24 h to obtain ~60% confluency. Stably transfected CHO-K1 cells were plated at 9 x 10^3^ cells/cm^2^ for pcDNA3.1 cells (clone A2) and 1.3 x 10^4^ cells/cm^2^ for hLCN2-R-FLAG isoform B cells (clone D6.D10) to compensate for differences in doubling time. Medium was replaced every 24 h with fresh 600 mosmol/l culture medium ± 5μg/ml LPS [32]. After treatments, cell viability was determined by the MTT test, as previously described [33].

### Cell death assays of rat primary IMCD cells

Lactate dehydrogenase release was measured using the CytoTox-Fluor cytotoxicity assay (Promega), according to manufacturer’s protocols.

### RNA extraction, cDNA synthesis and RT-PCR

Isolation of total RNA, synthesis of cDNA and PCR reactions were performed as previously described [34]. PCR reactions were performed using specific primers and cycling protocols (see Table 2). Gel documentation and densitometry analysis were performed using Image Lab Software version 5.2 (Bio-Rad Laboratories) with correction for loading with the housekeeping gene *Gapdh*.

**Table 2:** Protocols for reverse transcription semi-quantitative PCR

	*Gapdh*	*Ctnnb1* (β-catenin)	*Lcn2*	Lcn2-R *(Slc22a17/*24p3-R*)*	hLCN2-R *isoform B*
Accession number	NM_001289726.1	NM_007614.3	NM_008491.1	NM_021551.4	(NM_016609.4)
Forward primer (5‘→3‘)	AGGGCTCATGACCACAGT	TTAAACTCCTGCACCCACCAT	CCACCACGGACTACAACCAG	CAGCCACCTCCTAACCGCTGTG	GCCATTCGCCACTGCTAC
Reverse primer (5‘→3‘)	TGCAGGGATGATGTTCTG	AGGGCAAGGTTTCGAATCAA	AGCTCCTTGGTTCTTCCATACA	CTCCCACTAGGCTCAAAGGCTGCT	GGAGAAGAGCCCAAGGACAGA
Activation	5 min 95^o^C	5 min 95^o^C	5 min 95^o^C	5 min 95^o^C	5 min 95^o^C
Cycle number	22	27	30-32	27-28	22
Denaturation	30 sec 94^o^C	30 sec 94^o^C	30 sec 94^o^C	30 sec 94^o^C	30 sec 94°C
Annealing	30 sec 60^o^C	30 sec 60^o^C	30 sec 60^o^C	30 sec 60^o^C	30 sec 55°C
Extension	30 sec 72^o^C	30 sec 72^o^C	30 sec 72^o^C	30 sec 72^o^C	30 sec 72°C
Final Extension	7 min 72^o^C	7 min 72^o^C	7 min 72^o^C	7 min 72^o^C	10 min 72°C
PCR product (bp)	112	73	100	86	255

*bp* = base pairs

### Quantitative PCR of mIMCD3 cells

RNA extraction and cDNA synthesis were performed as described for the RT-PCR protocol. Resulting cDNA was diluted 1:10 with 1/10 TE buffer. Primers were designed using PrimerBLAST software (NCBI) and/or taken from the literature. The primers were obtained from Eurofins Genomics and primer efficiency was assessed using serially diluted mixed cDNAs (see Table 3). Quantitative PCR (qPCR) was performed essentially as previously described [35] in a StepOnePlus Real-Time PCR System (Applied Biosystems) using KAPA SYBR FAST qPCR Master Mix Universal and ROX reference dye (PEQLAB Biotechnologie). The cycling conditions were activation at 95 °C for 1 min, 40-50 cycles of 95 °C for 3 s and 60 °C for 30 s with melt curve analysis to check amplification specificity of the product. Gene expression levels were calculated according to the 2^*−ΔCq*^ method relative to the sample with the highest expression (minimum *Cq*) [36]. The data obtained were normalized to the expression of three stable reference genes: *Gapdh*, *Actb*, and *B2m*.

**Table 3:** qPCR primers

Gene-name (Accession number)	Forward primer (5‘→3‘)	Reverse primer (5‘→3‘)	Amplicon size (bp)	Efficiency (%)
*Actb* *(NM_007393.5)*	CGTGCGTGACATCAAAGAGAA	GGCCATCTC CTGCTCGAA	61	102
*Ctnnb1* (β-catenin*)* *(NM_001165902.1)*	TTAAACTCCTGCACCCACCAT	AGGGCAAGGTTTCGAATCAA	73	103
*B2m* *(NM_009735.3)*	AAATGCTGAAGAACGGGAAAA	ATAGAAAGACCAGTCCTTGCTGAAG	77	96
*Myc* *(NM_001177352.1)*	CGTTGGAAACCCCGCAGA	TACGGAGTCGTAGTCGAGGT	86	97
*Ccnd1* *(NM_007631.2)*	ACTGCCGAGAAGTTGTGCAT	AAGCAGTTCCATTTGCAGCAG	72	101
*Gapdh* *(NM_008084.3)*	CGGCCGCATCTTCTTGTG	CCGACCTTCACCATTTTGTCTAC	59	100
*Lcn2* *(NM_008491.1)*	CCACCACGGACTACAACCAG	AGCTCCTTGGTTCTTCCATACA	100	98
Lcn2-R *(Slc22a17)* *(NM_021551.4)*	CAGCCACCTCCTAACCGCTGTG	CTCCCACTAGGATCAAAGGCTGCT	86	110
*Wnt4* *(NM_009523.2)*	GAGAAGTGTGGCTGTGACCGG	ATGTTGTCCGAGCATCCTGACC	77	92
*Wnt5a* *(NM_009524.3)*	CTCCTTCGCCCAGGTTGTTATAG	TGTCTTCGCACCTTCTCCAATG	196	117

*bp* = base pairs

### Quantitative PCR of rat primary IMCD cells

Total RNA was isolated from rat primary IMCD cells using a RNeasy-kit (Qiagen) and cDNA was synthesized according to standard protocols using SuperScript II Kit (Invitrogen). Quantitative PCR (qPCR) was performed using SYBR Green PCR Master Mix on the ABI PRISM 7900 Sequence Detection System. All qPCR instruments and reagents were purchased from Thermo Fisher Scientific (Darmstadt, Germany). Relative gene expression values were evaluated with the 2^*−∆∆Ct*^ method using *Gapdh* as reference (ref PMID: 11846609). The primer pairs used were for rat *Gapdh* (GenBank accession number NM_017008.4): forward 5’-CATCAACGACCCCTTCATTGAC-3′, reverse 5’-ACTCCACGACATACTCAGCACC-3’ and for rat *Lcn2* (GenBank accession number NM_130741.1) forward 5’-GGGCTGTCGCTACTGGATCAG-3’, reverse 5’-CGTACAGGGTGACTTTGAAGTAC -3’.

### Determination of Lcn2 secretion

Lcn2 secretion by mIMCD3 and CHO-K1 cells was detected by immunoblotting using recombinant rodent Lcn2 to generate a standard curve. Supernatants from 2 wells of a 6 well plate were pooled (3 ml total volume) for each treatment and concentrated at room temperature (RT) to a final volume of 30-50 μl using Vivaspin 500 Centrifugal Concentrators (10 kDa molecular weight cut-off; cat. # VS0102, Sartorius). Volumes were adjusted, combined with 3X Laemmli buffer and used for immunoblotting. Lcn2 immunoblot signals from supernatants were normalized to cell numbers determined using a Countess II FL Automated Cell Counter (Thermo Fisher). Supernatants from stably transfected CHO-K1 cells were immunoblotted either non-concentrated or concentrated only by ~4-fold to avoid interference by FBS contamination.

Lcn2 in supernatants of primary rat IMCD cells was detected using the Rat Lcn2 DuoSet ELISA (R&D Systems) according to manufacturer’s protocol.

### Isolation of plasma membranes from mIMCD3 and CHO-K1 cells

1.25 x 10^6^ mIMCD3 cells were seeded into 175 cm^2^ culture flasks and grown for 24 h before medium was replaced by fresh normosmotic or hyperosmotic media and cultured for additional 72 h. Plasma membranes (PM) from transiently transfected CHO-K1 cells were obtained following G418 selection as previously described [16] with the following modification: After cell homogenization, unbroken cells, nuclei, and large debris were removed by centrifugation at 1000 x *g* for 10 min. PM enrichment was verified by immunoblotting and showed an ~20-fold enrichment in Na^+^,K^+^-ATPase.

### Immunoblotting

Sodium dodecyl sulfate polyacrylamide gel electrophoresis (SDS-PAGE) and immunoblotting were performed according to standard procedures by wet transfer, as described earlier [16], with the exception of Lcn2 immunoblots that were electroblotted by rapid semi-dry transfer (Bio-Rad Laboratories Trans-Blot Turbo). Cells were washed in PBS, scraped and homogenized by sonication (Branson Digital Sonifier) in isosmotic sucrose buffer supplemented with protease inhibitor cocktail. Protein concentration was determined by the Bradford method [37], using bovine serum albumin (BSA) as standard. For the analysis of phosphorylated proteins, cells were washed once with PBS and scraped into lysis buffer containing phosphatase inhibitors as previously described [38], and protein was determined by the method of Lowry *et al.* [39]. Equal amounts of protein (2.5-50 μg) were subjected to 6 or 15% SDS-PAGE and immunoblotted. Primary antibodies and their dilutions are listed in Table 1. For anti-phosphorylation and Lcn2 antibodies, dilution of primary antibodies was performed in 5% BSA. Horseradish peroxidase-conjugated secondary antibodies were purchased from Santa Cruz Biotechnology, Inc. or GE Healthcare and used at 1:2,500 – 1:30,000 dilutions. Densitometry analysis was performed using ImageJ software [40].

### Surface expression of Lcn2-R in rat primary IMCD cells by flow cytometry

Rat IMCD cells were dissociated using Accutase® solution (Sigma) and collected by centrifugation (5 min, 500 x *g*). The supernatant was discarded and cells were washed with PBS and centrifuged once more (5 min, 500 x *g*). The cell pellet was resuspended in 1% BSA in PBS (1% BSA-PBS) with anti-N-terminal rodent Lcn2-R polyclonal rabbit IgG (see Table 1) (1:200 dilution) and incubated for 45 min at RT. The cells were collected by centrifugation, washed twice with PBS and incubated for 30 min at RT with PBS containing a 1:500 dilution of secondary Alexa Fluor 488-conjugated goat anti-rabbit IgG. Cells were washed once in PBS, resuspended in FACSFlow buffer (BD Biosciences) and analyzed on a FACS Calibur flow cytometer (BD Biosciences) using the CellQuest Pro software (BD Biosciences).

### Immunofluorescence staining and microscopy

Immunofluorescence microscopy of mIMCD3 cells (1.0-4.0 x 10^4^ cells / well on glass coverslips, 40-50% confluency after 24 h) was either performed in non-permeabilized cells to detect surface expression of Na^+^/K^+^-ATPase and Lcn2-R, or in permeabilized cells to detect cellular Lcn2 and Lcn2-R expression. Surface staining was performed at 4^o^C whereas staining of permeabilized cells was carried out at RT.

For surface staining, cells were blocked with 1% BSA-PBS for 1 h before incubation with primary antibody in 1% BSA-PBS for 2 h. Cells were then incubated with secondary Cy3-coupled donkey anti-rabbit IgG (1:600; cat. # 711-165-152, Jackson ImmunoResearch) or Alexa Fluor 488-conjugated goat anti-rabbit IgG (1:500; cat. # A-11008, Thermo Fisher) for 1 hour. Cells were fixed with 4% paraformaldehyde (PFA) in PBS for 30 min at RT. Nuclei were counterstained with 0.8 μg/ml Hoechst 33342 for 5 min. Coverslips were embedded with DAKO fluorescent mounting medium, and images were acquired and fluorescence was quantified as described elsewhere [41].

For cellular Lcn2 or Lcn2-R staining, cells were fixed with 4% PFA in PBS for 10 min, permeabilized with 1% SDS in PBS for 5 min, and blocked with 1% BSA-PBS for 40 min. Primary antibodies diluted in 1% BSA-PBS were incubated for 2 h. Subsequent steps were identical to surface staining procedures.

For β-catenin siRNA transfected cells, 2500 cells/well were plated on glass coverslips in 24-well plates and grown for 72 h to reach a confluency of 30-40%. Immunostaining of permeabilized mIMCD3 cells for Lcn2-R was performed 48 h after transfection essentially as described above, but with the following modifications: Cells were fixed with 4% PFA in PBS for 30 min and permeabilized with 1% SDS in PBS for 20 min.

Immunofluorescence staining of rat primary IMCD cells was performed as previously described [42]. Immunofluorescence images were captured with an epifluorescence Axiovert Z1 microscope and a digital camera AxioCamMR (Carl Zeiss).

### Statistics

Unless otherwise indicated, the experiments were repeated at least three times with independent cultures. Means ± SEM are shown, unless otherwise indicated. Statistical analysis using unpaired Student's *t*-test was carried out with GraphPad Prism v. 5.01 (GraphPad Software Inc., San Diego California USA). Results with *P* < 0.05 were considered to be statistically significant. Significance levels were labelled in the Figures as follows: * = *P* < 0.05, ** = *P* < 0.025 and *** = *P* < 0.01.

## Results

### Hyperosmolarity increases expression of Lcn2-R in mIMCD3 and rat primary IMCD cells

**Fig. 1:**
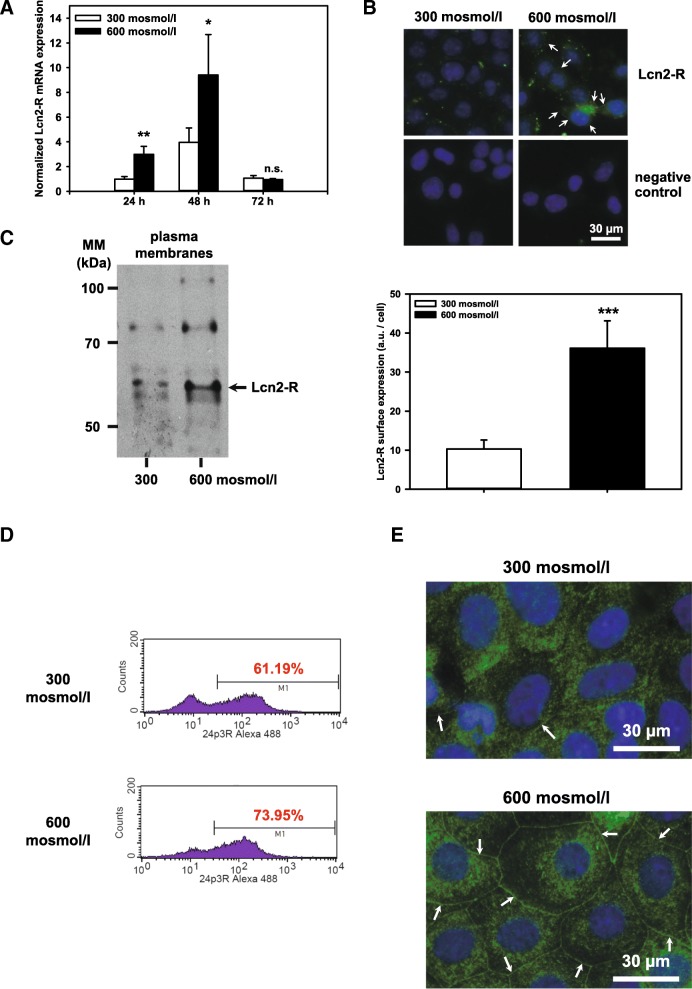
Hyperosmolarity increases Lcn2-R expression in mIMCD3 cells and primary rat IMCD cells. **a** Expression levels of Lcn2-R mRNA by qPCR in mIMCD3 cells exposed to 300 (normosmolarity/-tonicity) or 600 mosmol/l (hyperosmolarity/-tonicity) for 24 - 72 h. Means ± SEM of 4-5 experiments are shown. The data obtained were normalized to the expression of the reference genes glyceraldehyde-3-phosphate dehydrogenase (*Gapdh*), β-actin (*Actb*), and β2-microglobulin (*B2m*). Statistical analysis compares the two osmotic conditions by unpaired *t*-test. n.s. = not significant. **b** Surface expression of Lcn2-R in mIMCD3 cells exposed to norm- or hyperosmotic media for 72 h. Lcn2-R was detected by live immunofluorescence microscopy of non-permeabilized cells using a Lcn2-R antibody directed against the extracellular N-terminus. Nuclei were counterstained with Hoechst 33342. Arrows indicate punctate staining along the plasma membranes of mIMCD3 cells. Statistical analysis shows means ± SEM of 4 experiments and compares the two osmotic conditions by unpaired *t*-test. a.u. = arbitrary units. **c** Immunoblotting of plasma membranes (PM) of mIMCD3 cells grown for 72 h in norm- or hyperosmotic media. Lcn2-R is expressed in PM at the expected molecular mass of ~62 kDa. The experiment is representative of three similar ones. **d** Histograms of Lcn2-R surface expression in rat primary IMCD cells exposed to 300 or 600 mosmol/l for 7 days. Lcn2-R was determined by flow cytometry using a Lcn2-R antibody directed against the extracellular N-terminus. The experiment is representative of three similar ones. **e** Expression of Lcn2-R in rat primary IMCD cells cultured in norm- or hyperosmotic media for a total of 7 days. Lcn2-R was detected by immunofluorescence microscopy of permeabilized cells using an Lcn2-R antibody directed against the N-terminus. Arrows indicate Lcn2-R staining along the plasma membranes of rat primary IMCD cells. Nuclei were counterstained with DAPI. The experiment is representative of three similar ones.

Previous studies in rodent kidney have demonstrated highest abundance of Lcn2-R in the IMCD [16]. Hence, we hypothesized that Lcn2-R expression is linked to hyperosmolarity. qPCR of mIMCD3 cells exposed to 600 mosmol/l showed increased Lcn2-R mRNA expression at 24 and 48 h exposure, compared to normosmotic conditions (Fig. 1a). Surface expression of Lcn2-R in mIMCD3 cells was increased by 600 mosmol/l at 72 h (Fig. 1b, upper right panel), which was detected by staining of non-permeabilized cells using an antibody directed against the extracellular N-terminus of rodent Lcn2-R where the punctate distribution represents specific receptor expression sites and/or receptor clusters on cellular plasma membranes [31]. Accordingly, immunoblotting of mIMCD3 cells showed increased Lcn2-R in PM of cells exposed to 600 mosmol/l for 72 h (Fig. 1c) at the expected molecular mass of ~62 kDa [8] (higher molecular weight bands could represent N-glycosylated Lcn2-R [31]). Similarly, primary rat IMCD cells cultured for 1 week in 600 mosmol/l showed increased surface expression of Lcn2-R, as determined by flow cytometry analysis of dissociated cells (Fig. 1d, bottom) and was verified by immunofluorescence microscopy of permeabilized primary rat IMCD cells (Fig. 1e, bottom), confirming induction of Lcn2-R gene expression. Similar observations were made for 900 mosmol/l media (*data not shown*). In contrast, Lcn2-R expression remained low under normosmotic conditions (Fig. 1) (see also model in Fig. 7a). Taken together, these data are consistent with *in vivo* medullary localization of Lcn2-R in the rat kidney [16] where it is elevated in a hyperosmotic environment.

### Hyperosmolarity decreases expression of Lcn2 in mIMCD3 and rat primary IMCD cells

**Fig. 2:**
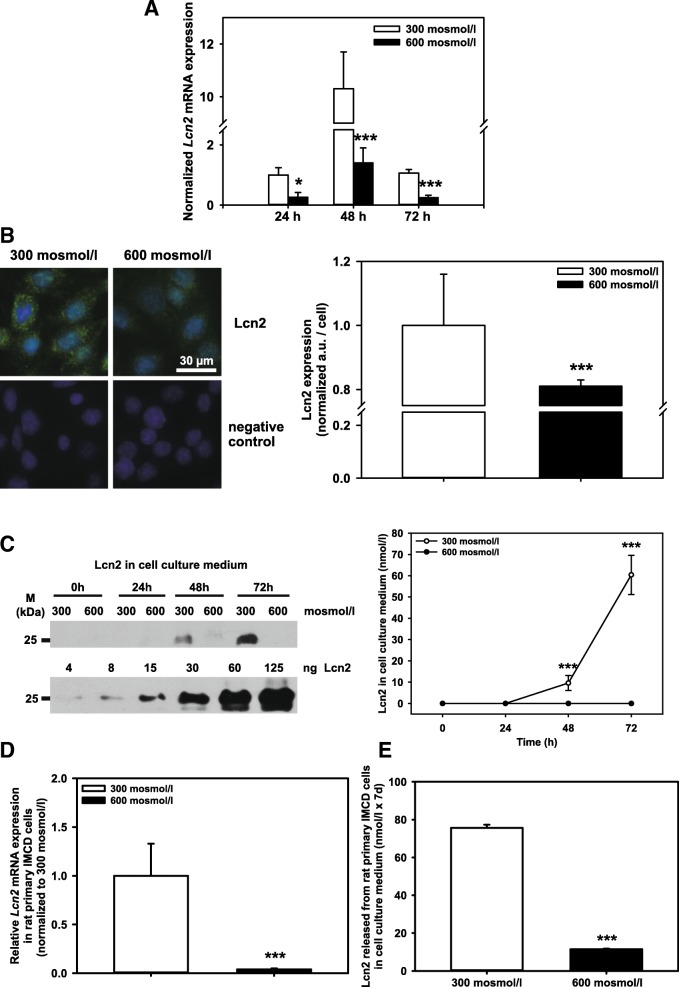
Hyperosmolarity decreases Lcn2 expression in mIMCD3 cells and rat primary IMCD cells. **a** Expression levels of *Lcn2* mRNA by qPCR in mIMCD3 cells exposed to 300 or 600 mosmol/l for 24-72 h. The data obtained were normalized to the expression of the reference genes glyceraldehyde-3-phosphate dehydrogenase (*Gapdh*), β-actin (*Actb*), and β2-microglobulin (*B2m*). Means ± SEM of 4-5 experiments are shown. Statistical analysis compares the two osmotic conditions by unpaired *t*-test. **b** Expression of Lcn2 in mIMCD3 cells exposed to norm- or hyperosmotic media for 72 h was detected by immunofluorescence microscopy of permeabilized cells. Nuclei were counterstained with Hoechst 33342. Statistical analysis shows means ± SEM of 3 experiments. Arbitrary units (a.u.) are normalized to normosmotic media (5.0 ± 1.1 a.u.), and the two osmotic conditions are compared by unpaired *t*-test. **c** Kinetics of Lcn2 release from mIMCD3 cells exposed to norm- or hyperosmotic media for 72 h. Lcn2 release was determined by immunoblotting. The upper blot is representative of 4 different experiments. To quantify experiments, Lcn2 standards were immunoblotted (lower blot). All immunoblot bands were analyzed by densitometry using ImageJ software and normalized to cell numbers. Lcn2 release (nmol/l) is plotted as means ± SEM of 4 experiments. Statistical analysis compares the two osmotic conditions by unpaired *t*-test. **d** *Lcn2* gene expression in rat primary IMCD cells cultured in norm- or hyperosmotic media for 7 days. Relative *Lcn2* gene expression was determined by qPCR. Means ± SD of 3 experiments are plotted. Data are normalized to 300 mosmol/l media and compares the two osmotic conditions by unpaired *t*-test. **e** Lcn2 release from rat primary IMCD cells one week after exposure to norm- or hyperosmotic media. Lcn2 in cell culture medium was assayed by ELISA. Means ± SEM of 7 experiments are shown and statistical analysis by unpaired *t*-test compares the two experimental conditions.

Next, the expression of the natural ligand of Lcn2-R, Lcn2 [8], was investigated under physiological, i.e. hyperosmotic, conditions for IMCD cells. Contrary to Lcn2-R, *Lcn2* mRNA expression was low in mIMCD3 cells exposed to hyperosmolarity for 24 – 72 h (Fig. 2a) but was increased at all time points investigated in normosmotic media (Fig. 2a). This was confirmed at the protein level by immunofluorescence microscopy of permeabilized cells (Fig. 2b, upper left panel; punctate staining likely represents intracellular vesicles). Paracrine/autocrine signaling is integral to Lcn2 effects therefore Lcn2 secretion was probed by immunoblotting of cell supernatants. At 48 h and 72 h, Lcn2 was detected in supernatants from mIMCD3 cells exposed to normosmotic media that progressively increased, implying continuous secretion, as opposed to cells in hyperosmotic media where no Lcn2 release into the medium was detected despite correction for cell number (Fig. 2c). These data were confirmed in rat primary IMCD cells where increased osmolarity for 1 week almost abolished *Lcn2* mRNA expression (Fig. 2d) and Lcn2 release (Fig. 2e). Thus, reminiscent of observations made in other cell systems [8, 9], an inverse co-regulation of Lcn2 and Lcn2-R is governed by osmotic changes in IMCD cells (see also models in Fig. 7a and c).

### Apo-Lcn2 induces death of Lcn2-R expressing primary IMCD cells exposed to hyperosmolarity

**Fig. 3:**
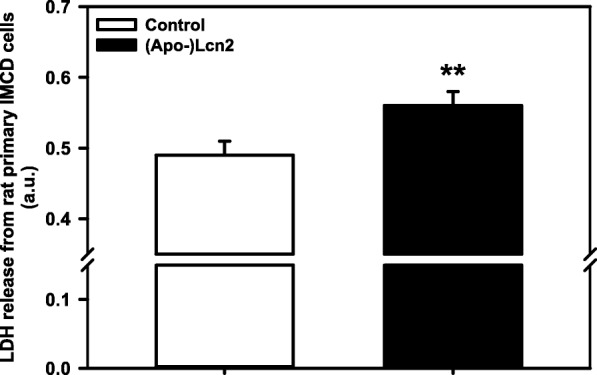
Apo-Lcn2 induces death of rat primary IMCD cells exposed to hyperosmolarity. Cells were exposed to hyperosmotic media for 7 days to increase Lcn2-R expression and incubated with 15 μg/ml apo-Lcn2 for 24 h. Cell death was determined by measuring lactate dehydrogenase (LDH) release into the medium. Means ± SEM of 4-6 experiments are shown and statistical analysis compares control versus apo-Lcn2 incubated cells by unpaired *t*-test. a.u. = arbitrary units

Several studies have demonstrated that Fe^3+^-free (apo-)Lcn2 induces death of Lcn2-R expressing cells [8, 9]. This hypothesis was further tested in primary IMCD cells that expressed elevated Lcn2-R due to exposure to hyperosmotic media for 7 days (see Fig. 1d). Co-incubation with 15 μg/ml recombinant rat apo-Lcn2 increased cell death, as indicated by augmented LDH leakage (Fig. 3). This Lcn2 concentration is ca. 10^4^ times higher than physiological urinary LCN2 concentrations in humans [43] and ~20-fold higher than urinary Lcn2 measured in patients with acute UTI [14]. However, apo-Lcn2 may be competing with other components of the culture medium, such as albumin or transferrin, which are also ligands of the Lcn2-R [16], thus diminishing the efficacy of recombinant apo-Lcn2. These data indicate that (apo-)Lcn2 mediates death of cells expressing the Lcn2-R, which is in line with previous concepts [44] and suggest that decreased expression of apo-Lcn2 when Lcn2-R is expressed may serve as a protective measure of IMCD cells against potential toxicity of autocrine/paracrine signaling of apo-Lcn2 in a physiological hyperosmotic environment (Figs. 1d-e, 2d-e) (see also model in Fig. 7b).

### Hyperosmolarity-induced Wnt/β-catenin signaling mediates differential expression of Lcn2 and Lcn2-R in mIMCD3 cells

The following lines of evidence suggested implication of Wnt/β-catenin signaling in the hyperosmotic response: Wnt-1 transfected mammary epithelial cells display decreased *Lcn2* expression and induction of a full-length transcript of Lcn2-R [25, 26], increased osmolarity activates Wnt signaling [23, 24], and osmolarity-induces an increase of *Myc*, a Wnt target gene (Additional file 1: Figure S1).

**Fig. 4:**
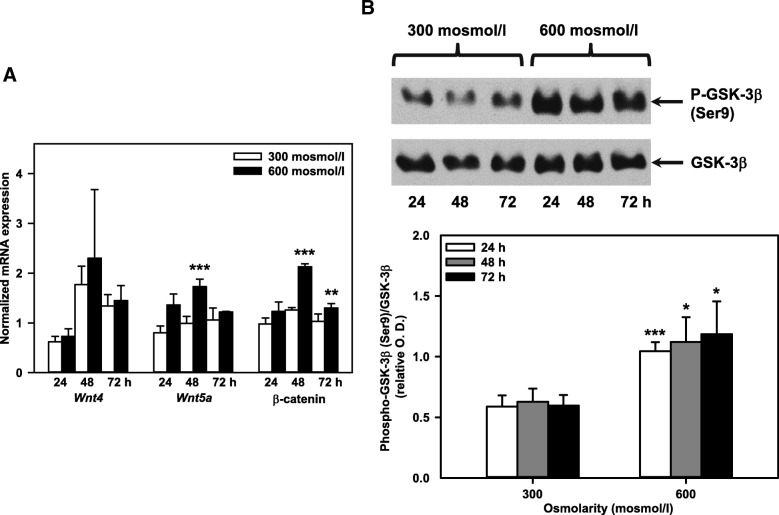
Hyperosmolarity increases Wnt/β-catenin signaling in mIMCD3 cells. **a** Expression levels of *Wnt4*, *Wnt5a* and β-catenin mRNA in mIMCD3 cells exposed to norm- or hyperosmotic media for 24-72 h measured by qPCR. The data obtained were normalized to the expression of the reference genes glyceraldehyde-3-phosphate dehydrogenase (*Gapdh*), β-actin (*Actb*), and β2-microglobulin (*B2m*). Means ± SEM of 3-4 experiments are shown. Statistical analysis compares the two osmotic conditions by unpaired *t*-test. **b** Expression of GSK-3β and phospho-GSK-3β (Ser9) in mIMCD3 cells was detected by immunoblotting. Optical density (O.D.) of phospho-GSK-3β (Ser9) and GSK-3β signals was analyzed by densitometry using ImageJ software, and relative O.D. was calculated from the ratio of phospho-GSK-3β (Ser9) / GSK-3β. Statistical analysis shows means ± SEM of 4 experiments and compares the two osmotic conditions by unpaired *t*-test.

Since Wnt ligands *Wnt 4* and *Wnt5a* are highly expressed in mouse kidney [45], we tested the effect of hyperosmolarity on these Wnt ligands in mIMCD3 cells: *Wnt5a*, but not *Wnt4* mRNA was significantly increased by hyperosmotic media for 48 h (Fig. 4a). Furthermore, increased osmolarity was associated with increased β-catenin mRNA at 48 h (Fig. 4a) that precedes *Myc* induction at 72 h (Additional file 1: Figure S1). Activation of the canonical Wnt signaling inhibits the kinase glycogen synthase kinase 3β (GSK-3β) in the β-catenin destruction complex, thereby preventing β-catenin phosphorylation and its proteasomal degradation [46]. Interestingly, GSK-3β phosphorylation at Ser9, which inactivates the kinase, was increased after exposure to hyperosmotic media for 24-72 h (Fig. 4b), confirming a previous study with HEK293 and mIMCD3 cells exposed to high NaCl, in which inhibitory phosphorylation of GSK-3β at Ser9 (GSK-3β-S9) activated TonEBP/OREBP/NFAT5 [47].

**Fig. 5:**
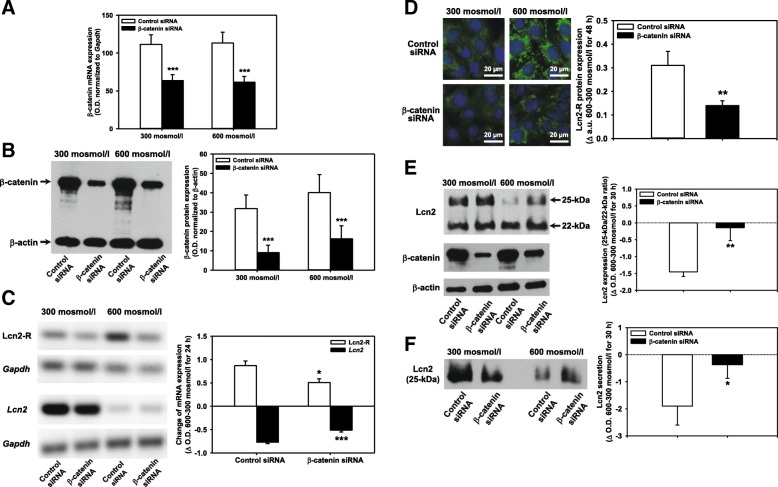
Silencing of β-catenin blunts alterations of Lcn2 and Lcn2-R expression induced by hyperosmolarity in mIMCD3 cells. **a** Following transfection with siRNA against β-catenin or control siRNA for 6 h, expression levels of β-catenin mRNA were determined by PCR in mIMCD3 cells exposed to norm- or hyperosmotic media for 24 h. The optical density (O.D.) of the signals was analyzed by densitometry using ImageJ software and β-catenin mRNA expression was normalized to the reference gene *Gapdh*. Means ± SEM of 11 experiments are shown. Statistical analysis compares the effects of control versus β-catenin siRNA by unpaired *t*-test. **b** Under the same experimental conditions and exposure to norm- or hyperosmotic media for 48 h, β-catenin protein expression was detected by immunoblotting. Expression was normalized by calculating the ratio of β-catenin to the loading control β-actin. Statistical analysis shows means ± SEM of 6 experiments and compares the effects of control versus β-catenin siRNA by unpaired *t*-test. **c** RT-PCR analysis of Lcn2-R and *Lcn2* mRNA in mIMCD3 cells transfected with control or β-catenin siRNA as described in (**a**). Lcn2-R and *Lcn2* mRNA expression was normalized to the reference gene *Gapdh*. The change of gene expression of Lcn2-R and *Lcn2* induced by osmotic stress (Δ O.D. 600-300 mosmol/l for 24 h) without or with β-catenin siRNA was plotted as means ± SEM of 3-5 experiments. Statistical analysis compared β-catenin silencing to control siRNA by unpaired *t*-test. **d** Effect of β-catenin silencing on the expression of Lcn2-R protein in mIMCD3 cells. Lcn2-R protein was detected by immunofluorescence microscopy of permeabilized cells. Nuclei were counterstained with Hoechst 33342. Statistical analysis determines the effect of β-catenin silencing on the change of Lcn2-R expression induced by osmotic stress (Δ a.u. 600-300 mosmol/l for 48 h) by unpaired *t*-test. Means ± SEM of 5 different experiments are shown. a.u. = arbitrary units. **e** Effect of β-catenin silencing on the expression of non-glycosylated ~22-kDa precursor and glycosylated ~25-kDa mature Lcn2 in mIMCD3 cells exposed to norm- or hyperosmotic media for 30 h. Statistical analysis determines the effect of β-catenin silencing on the reduction of secretory Lcn2 protein in cells (ratio of 25 to 22-kDa Lcn2) induced by osmotic stress (Δ O.D. 600-300 mosmol/l for 30 h) using unpaired *t*-test. Means ± SEM of 5 different experiments are plotted. **f** Effect of β-catenin silencing on Lcn2 protein secretion into culture media. Lcn2 was detected by immunoblotting as a 25-kDa band (see Additional file 4: Figure S4C). Signals were analyzed by densitometry using ImageJ software and normalized to cell numbers. Statistical analysis determines the effect of β-catenin silencing on the decrease of secreted Lcn2 elicited by osmotic stress (Δ O.D. 600-300 mosmol/l for 30 h) by unpaired *t*-test. Means ± SEM of 5 experiments are plotted.

To determine whether Wnt/β-catenin signaling is causally involved in the osmolarity-dependent regulation of Lcn2 and Lcn2-R expression, mIMCD3 cells were transfected with siRNA against β-catenin for 6 h prior to exposure to hyperosmotic media, which downregulated β-catenin mRNA after 24 h by ~45% (Fig. 5a; Additional file 3: Figure S3A), that was stable for up to 48 h (see Additional file 3: Figure S3B), and β-catenin protein after 24-48 h by ~65% (Fig. 5b and e). Silencing of β-catenin blunted Lcn2-R up- and *Lcn2* downregulation elicited by high osmolarity at the mRNA level (Fig. 5c). Similarly, the increase of Lcn2-R protein induced by high osmolarity was significantly attenuated by β-catenin silencing, as determined by immunofluorescence microscopy of mIMCD3 cells (Fig. 5d, compare upper and lower right panels). Analogous to neutrophils, two bands for Lcn2 were detected in whole mIMCD3 cell homogenates that represent the non-glycosylated ~22-kDa precursor and glycosylated ~25-kDa mature Lcn2 [6, 11]. Only the latter was found to be secreted by mIMCD3 cells upon hyperosmolarity (Fig. 2c) or stimulation by the bacterial wall component LPS [14, 32] (Additional file 4: Figure S4C, lane 2). Increased osmolarity had a larger inhibitory effect on mature Lcn2 formation which was almost completely reversed by β-catenin silencing (Fig. 5e, lanes 3 and 4). Little effect was seen on the precursor. Moreover, β-catenin siRNA significantly restored Lcn2 secretion in hyperosmotic media (Fig. 5f, lanes 3 and 4). In conclusion, hyperosmolarity engages Wnt/β-catenin signaling to inversely regulate Lcn2 and Lcn2-R (see also models in Fig. 7).

### LPS interferes with changes of Lcn2-R and Lcn2 expression/secretion induced by hyperosmolarity in mIMCD3 cells

**Fig. 6:**
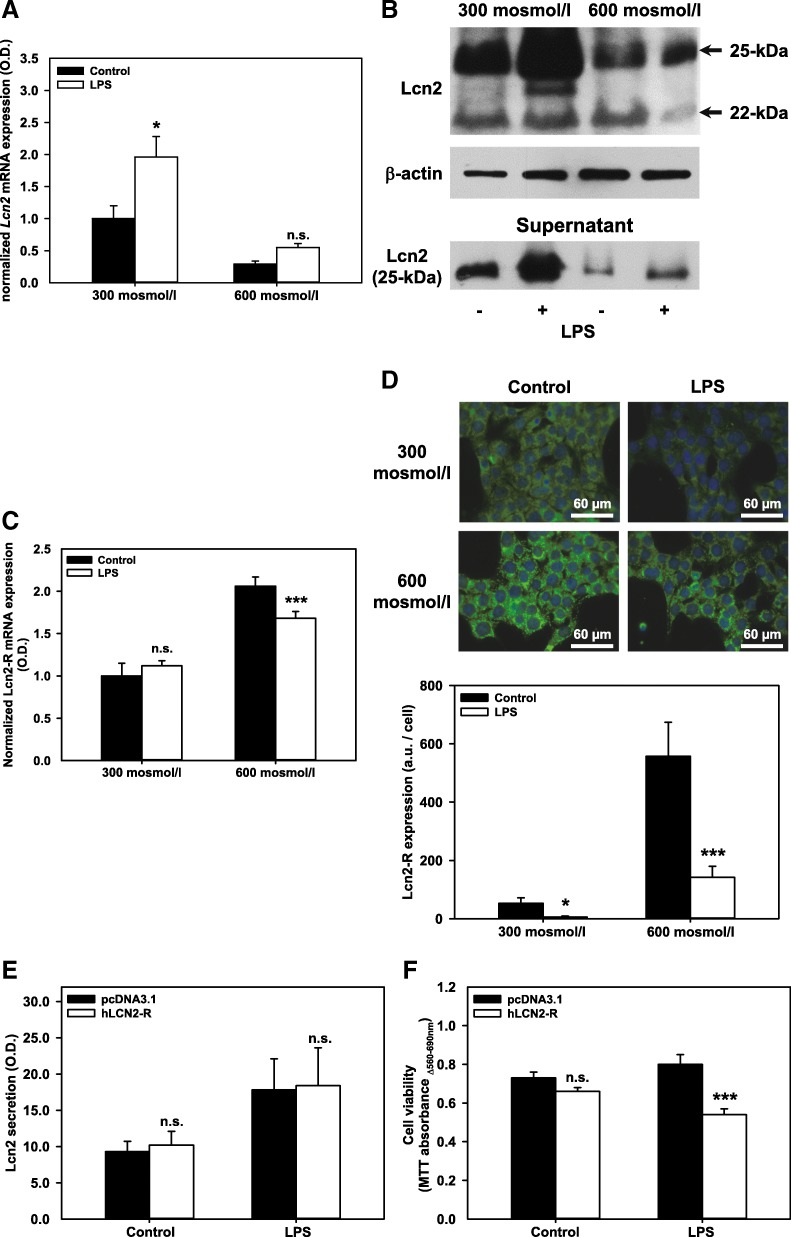
LPS reverts hyperosmolarity-induced changes of Lcn2-R/Lcn2 expression in mIMCD3 cells and decreases viability of CHO-K1 cells stably overexpressing hLCN2-R. mIMCD3 cells were exposed to norm- or hyperosmotic media for 24 h and ± LPS for additional 18 h. **a** RT-PCR analysis of *Lcn2* mRNA, which was normalized to the reference gene *Gapdh*. *Lcn2* gene expression, was plotted as means ± SEM of 4 experiments. Statistical analysis determines the effect of LPS by unpaired *t*-test. n.s. = not significant. **b** Effect of LPS (5 μg/ml for 18 h) on the cellular expression of 22-kDa precursor, 25-kDa mature Lcn2, and secretion of 25-kDa Lcn-2 in mIMCD3 cells. The experiment is representative of three similar ones. **c** Lcn2-R mRNA expression was determined by RT-PCR and normalized to the reference gene *Gapdh*. Means ± SEM of 6 experiments are shown. Statistical analysis compares LPS to control by unpaired *t*-test. n.s. = not significant. **d** Effect of LPS on the expression of Lcn2-R in mIMCD3 cells exposed to norm- or hyperosmotic media for 24 h and ± LPS (5 μg/ml) for additional 18 h. Lcn2-R protein was detected by immunofluorescence microscopy of permeabilized cells. Nuclei were counterstained with Hoechst 33342. Lcn2-R protein expression at 300 and 600 mosmol/l ± LPS was plotted as means ± SEM of 4 experiments. Statistical analysis determines the effect of LPS by unpaired *t*-test. a.u. = arbitrary units. **e** Both, CHO-K1 cell clones stably transfected with pcDNA3.1 or human lipocalin-2 receptor isoform B (hLCN2-R), when grown in hyperosmotic media secrete similar amounts of Lcn2 upon treatment with LPS (5 μg/ml for 48 h). Secretion of Lcn2 was determined by immunoblotting. The optical density (O.D.) of Lcn2 signals was quantified by densitometry using ImageJ software. Means ± SEM of 4 experiments are plotted. **f** Cell viability of both clones stably transfected with pcDNA3.1 or hLCN2-R was measured by MTT assay. Data show means ± SEM of 5-6 experiments with 6 replicates per experiment. Statistical analysis compares conditions ± hLCN2-R using unpaired *t*-test; n.s. = not significant.

**Fig. 7:**
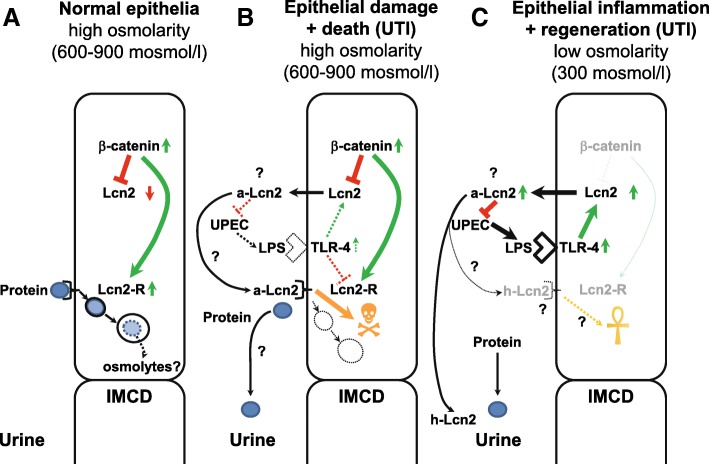
Model of the effects of osmolarity, Wnt/β-catenin, and TLR-4 signaling on the expression of the Lcn2-R and its ligand Lcn2 in IMCD cells of the kidney. **a** Under physiological conditions and high interstitial osmolarity, Wnt/β-catenin signaling is activated which leads to downregulation of Lcn2 and upregulation of the Lcn2-R. The latter binds proteins in the primary urine with high affinity that are degraded in lysosomes, possibly to generate adaptive osmolytes. **b** Bacterial infection in an initially hyperosmotic environment induces TLR-4 signaling that counteracts Wnt/β-catenin signaling, leading to disinhibition of Lcn2 expression and secretion. Urinary Lcn2 displaces proteins from the Lcn2-R as long as it is expressed in the apical membrane, and Lcn2 endocytosis leads to death of IMCD cells. **c** Prolonged bacterial infection amplifies TLR-4 signaling and (apo-)Lcn2 secretion to combat UTI by binding bacterial siderophores and excretion of holo-Lcn2 into the urine along with proteins. Cell death leads to inflammation and a decrease of interstitial osmolarity, which downregulates Wnt/β-catenin signaling, further amplifying Lcn2 expression and secretion while downregulating Lcn2-R expression. Lcn2-R that may still be present in the luminal membrane may take up siderophore-bound holo-Lcn2 to prevent cell death and contribute to epithelial regeneration. For further details, see “Results” and “Discussion”. The thickness of the arrows reflects the strength of gene expression, activity and/or signaling pathways. Dotted lines and shading indicate weak expression and/or signals. Question marks indicate putative mechanisms that have not been tested in this study and are addressed in the discussion. Lcn2 = lipocalin-2; a-Lcn2 = apo-Lcn2; h-Lcn2 = holo-Lcn2; Lcn2-R = lipocalin-2 receptor; IMCD = inner medullary collecting duct; UTI = urinary tract infection; UPEC = uropathogenic E- coli; LPS = lipopolysaccharide; TLR-4 = Toll-like receptor-4.

So far, we have evidenced that mIMCD3 cells increase Lcn2-R expression and suppress Lcn2 expression and secretion in a physiological high osmolarity context, which is regulated by Wnt/β-catenin signaling. The role of these mechanisms in a pathophysiological context was then explored, namely infection of the distal nephron by uropathogenic E. coli (UPEC), which represents the primary cause of UTI, including both cystitis and pyelonephritis [48]. To mimic bacterial infection by UPEC, we applied LPS to mIMCD3 cells in a hyperosmotic environment, which has been shown to elicit secretion of Lcn2 in kidney medulla [14]. Similarly as described in Fig. 2, exposure of mIMCD3 cells to hyperosmotic media decreased expression of *Lcn2* mRNA (Fig. 6a and Additional file 4: Figure S4D, compare lanes 1 and 3) as well as expression of mature (~25 kDa) Lcn2 protein and its secretion (Fig. 6b). Likewise, Lcn2-R *mRNA* and protein expression were upregulated (Fig. 6c and Additional file 4: Figure S4E, compare lanes 1 and 3; Fig. 6d, compare upper and lower left panels) after 42 h or 72 h in hyperosmotic media, which recapitulates Fig. 1. Treatment with a saturating concentration of LPS (5 μg/ml) [32] (see Additional file 4: Figure S4B) during the last 18 h significantly reversed these effects, that is Lcn2 expression and secretion were partially restored (Fig. 6a and b, lane 4; Additional file 4: Figure S4D, lane 4) and Lcn2-R elevation was diminished (Fig. 6c and Additional file 4: Figure S4E, lane 4; Fig. 6d, compare lower left and right panels). Shorter LPS treatment periods than 18 h did not yield significant Lcn2 secretion in normosmotic media (Additional file 4: Figure S4A). These data demonstrate that LPS mediated signaling counteracts hyperosmolarity induced regulation of Lcn2 and Lcn2-R expression in IMCD cells. Moreover, decreasing osmolarity to 300 mosmol/l strongly enhanced LPS-induced Lcn2 expression and secretion (Fig. 6a and b, lanes 1 and 2) (see also models in Fig. 7b and c).

### LPS-induced Lcn2 secretion decreases viability of human Lcn2-R overexpressing CHO-K1 cells

The role of the Lcn2-R in induction of cell death during LPS-induced Lcn2 secretion was further investigated in CHO-K1 cells with and without stable transfection of hLCN2-R isoform B and incubated in hyperosmotic media. Although comparable amounts of Lcn2 secretion was induced in both CHO-K1 cell clones by incubation with LPS (5 μg/ml for 48 h) (Fig. 6e), only CHO-K1 cells overexpressing hLCN2-R isoform B showed significant reduction in cell viability upon LPS treatment compared to pcDNA3.1 transfected cells (Fig. 6f).

Overall, these data suggest that LPS and/or reduced osmolarity increase the release of bacteriostatic Fe^3+^-free apo-Lcn2 into the urine and thus contribute to fighting bacterial UTIs, whereas downregulation of Lcn2-R may protect IMCD cells against damage and death induced by released apo-Lcn2 (see also models in Figs. 7b and c).

## Discussion

The study demonstrates that in a hyperosmotic environment, as encountered *in vivo* under physiological conditions in the kidney medulla, IMCD cells upregulate Lcn2-R and downregulate Lcn2, whereas the opposite is the case under normotonic conditions. Both Lcn2 and Lcn2-R are downstream target genes of Wnt/β-catenin signaling that may be activated as an adaptive measure to stress elicited by hyperosmolarity. The inverse regulation of Lcn2-R and Lcn2 by Wnt/β-catenin signaling may be relevant to prevent cell damage and death by Lcn2 in a hyperosmotic environment and may be reversed during tubulo-interstitial diseases involving inflammation, e.g during UTI, where a normosmotic environment may more efficiently combat against bacterial infections (see Fig. 7c and below).

Increased Wnt/β-catenin signaling was observed in mIMCD3 cells exposed to hyperosmotic media with increased expression of *Wnt5a* and β-catenin mRNA (Fig. 4a), suggesting an adaptive process. Induction of Wnt/β-catenin signaling has not been described so far as an adaptive response to hyperosmotic stress in IMCD cells, but precedents exist from other cell types [23, 24]. Interestingly, increased Wnt/β-catenin signaling paralleled a second phase of expression of cell cycle genes *Myc* and *Ccnd1* that were upregulated or restored after 72 h (Additional file 1: Figure S1B). Both *Myc* and *Ccnd1* are downstream target genes of the β-catenin/TCF transcription factor complex [49–51], and this induction could contribute to reactivation of proliferation in adapted cells.

Wnt/β-catenin signaling seems to support and/or complement TonEBP/OREBP/NFAT5-mediated adaptive signaling. Whether the latter pathway is up- or downstream from Wnt/β-catenin signaling, or represents a parallel pathway is currently unclear. The osmoprotective master regulator of hyperosmolarity TonEBP/OREBP/NFAT5 appears to represent an upstream activator of canonical Wnt signaling, as evidenced during cardiomyogenic differentiation [52]. In contrast, a study in intestinal cells suggested that TonEBP represses Wnt/β-catenin signaling and thereby promotes enterocyte differentiation [53], but these may be cell-type specific effects unrelated to osmolarity induced cell proliferation and differentiation. Conversely, another study in HEK293 and mIMCD3 cells showed inhibitory phosphorylation of GSK-3β (GSK-3β-S9) by AKT, PKA, and PI3K contributing to high NaCl-induced activation of TonEBP/OREBP/NFAT5, and suggests upstream regulation of TonEBP by Wnt/GSK-3β signaling [47]. Hence, it would be intriguing to assess the impact of osmotic stress-induced TonEBP/OREBP/NFAT5 signaling on regulation of Lcn2 and Lcn2-R expression.

A previous *in vivo* study in mouse has demonstrated a link between osmolarity and Lcn2 expression. Aquaporin-1 null mice lack a functional countercurrent multiplier mechanism and fail to concentrate the inner medullary interstitium. In these mice, *Lcn2* mRNA expression was increased in the inner medulla [54], which is in line with the data shown in Fig. 2. It is interesting that the ~22-kDa Lcn2 precursor was not affected by increased osmolarity (Fig. 5e, lanes 1 and 3) although gene expression was decreased (Figs. 2 and 5c, lanes 1 and 3). Still, the total amount of Lcn2 protein (~22-kDa + ~25-kDa) as well as the ratio of mature to precursor Lcn2 protein were also reduced (Figs. 2 and 5e), which suggests that high osmolarity influences both Lcn2 gene expression and trafficking/maturation.

No *in vivo* studies have been performed so far that would support our observations showing the upregulation of Lcn2-R by increased osmolarity (Fig. 1). Yet our previous data showing high Lcn2-R expression in normal kidney medulla in rodents [16] as well as recent data obtained by deep sequencing in microdissected rat IMCD where in Lcn2-R had the highest expression from all nephron segments and *Lcn2* levels were negligible [55] support our observations. Moreover, progressive kidney tubulo-interstitial damage (e.g. due to recurring bacterial infections, renal ischemia, or proteinuric nephropathies), which impairs the renal capacity to concentrate urine [1, 56, 57], results in reduction of Lcn2-R expression [17], concomitantly with an increase of Lcn2 expression ([14] and this study).

The inverse regulation of Lcn2 and Lcn2-R induced by osmolarity via Wnt/β-catenin signaling is not unique to renal medullary cells and has been reported in other cell types. Previous studies in cancer cells have already demonstrated an inverse relationship between Lcn2 and Lcn2-R expression. In murine myeloblast-like cells, the oncogene BCR-ABL activated the JAK/STAT pathway, which increased Lcn2, and repressed Lcn2-R expression in a Ras and Runx1-dependent manner [8, 9].

Although other signaling pathways likely regulate Lcn2 and Lcn2-R expression, the contribution of β-catenin was strong, accounting for ~50% of Lcn2-R expression (Fig. 5c and d) and more than 80% of Lcn2 expression and secretion (Fig. 5e and f) despite incomplete β-catenin silencing (Fig. 5 and Additional file 3: Figure S3). These results support findings from Ziegler *et al.* showing that the Wnt/β-catenin pathway inhibits the *Lcn2* promoter [25, 26]. Although the study by Ziegler *et al.* only showed Wnt/β-catenin-dependent differential splicing of Lcn2-R transcripts [25], *in silico* analysis supports the hypothesis that Lcn2-R is a downstream target of Wnt/β-catenin signaling. Putative transcription factor binding sites in the DNA sequence flanking murine Lcn2-R (*Slc22a17*) were analyzed [58, 59] wherein a putative TCF1 binding site was found 111 bases upstream of the *Slc22a17* gene indicating a possible binding domain in the promoter region and supporting the hypothesis that Lcn2-R is a direct target gene of Wnt/β-catenin signaling.

The significance of upregulation of Lcn2-R in a physiological hyperosmotic environment is difficult to test as long as kidney-specific knockout animals are not available. We have previously demonstrated that Lcn2-R is involved in high-affinity protein endocytosis in the distal nephron [16] and suggested that the Lcn2-R could contribute to clearance of filtered proteins by the nephron under physiological conditions. Support for the hypothesis that Lcn2-R may endocytose luminal proteins in the distal nephron has been recently provided for its natural ligand Lcn2 [60]. Endocytosed proteins are trafficked to lysosomes [16] where they are degraded. It is therefore not unreasonable to speculate that the Lcn2-R may contribute to adaptation to osmotic stress by providing amino acids as precursors/osmolytes to IMCD cells to maintain isoosmolarity with the interstitium. But this remains to be proven. Prevention of Lcn2 expression and secretion, on the other hand, would also avert death-promoting effects of (apo-)Lcn2 in cells that are already osmotically stressed (Figs. 3 and 6).

The ligand of Lcn2-R, Lcn2 [19, 31], is secreted by the renal CD [12, 14] where it contributes to limitation of tubular stress and injury caused by a number of insults, e.g. by exerting bacteriostatic effects [61, 62]. Parallel expression/secretion of Lcn2-R and Lcn2 under conditions of renal medullary hyperosmolarity, which may be encountered during early stages of bacterial infection, would be deleterious for two reasons: 1) autocrine or paracrine endocytosis of secreted (apo-)Lcn2 would promote cell damage [8] (see also Figs. 3 and 6) rather than limit the insult; and 2) endocytosis of Lcn2 via Lcn2-R would reduce the availability of Lcn2 in the lumen and thereby reduce the siderophore-Fe^3+^ scavenging and bacteriostatic effect of apo-Lcn2. Downregulation of Lcn2-R as well as upregulation and increased secretion of Lcn2 induced by an isosmotic interstitium and/or LPS, which may be encountered during inflammation-associated hyperperfusion and/or late reparative stages of bacterial infection [56, 57], could offset these detrimental consequences (see Fig. 7c). LPS-induced activation of the TLR-4 pathway [14] opposes TonEBP/OREBP/NFAT5 signaling [63] and could possibly also counteract Wnt/β-catenin signaling induced by hyperosmolarity.

Of course, the consequences and outcome would be entirely different in case of the presence of siderophore Fe^3+^-loaded holo-Lcn2 in the tubular lumen that is known to promote repair, survival and proliferation of normal [64] and cancerous cells [65]. In this case, co-expression/secretion of Lcn2-R and holo-Lcn2 would be desirable for epithelial proliferation and regeneration (see Fig. 7c). Sporadic evidence for this hypothesis has been obtained from cancer studies (reviewed in [10]), but systemic studies investigating the role of apo- versus holo-Lcn2 in renal epithelial regeneration following various insults would be desirable. On the other hand, it is interesting to note that we have previously obtained evidence that siderophore Fe^3+^-loaded human holo-LCN2 binds with lower affinity to the N-terminus of human LCN2-R than apo-LCN2 [31]. Though speculative, this observation could signify that luminal holo-LCN2 may be more dedicated to excretion into the urine as an effective bacteriostatic measure to dispose of luminal iron rather than to auto- or paracrine uptake (see Fig. 7b and c) .

## Conclusions

In summary, Lcn2-R up- and Lcn2 downregulation via Wnt/β-catenin may promote adaptive survival of IMCD cells in response to hyperosmolarity whereas Lcn2 up- and Lcn2-R downregulation via LPS/TLR-4 and/or normosmolarity may protect IMCD cells against bacterial infections and prevent autocrine death induction by secreted (apo-)Lcn2.

## Additional files

Additional file 1: **Figure S1.** Hyperosmolarity inhibits proliferation and induces adaptive responses in mIMCD3 cells. (**A**) Proliferation of mIMCD3 cells cultured in media without (normosmolarity) or with 100 mmol/l urea and NaCl (hyperosmolarity) for 72 h. Measurements of MTT absorbance/optical density (O.D.) reflecting cell proliferation were performed at indicated time points. Means ± SEM of 5 experiments are shown. Statistical analysis compares the two osmotic conditions at each time point by unpaired *t*-test. (**B**) Expression levels of proliferation genes c-Myc (*Myc*) and cyclin D1 (*Ccnd1*) by qPCR in mIMCD3 cells exposed to norm- or hyperosmotic media for 72 h. The data obtained were normalized to the expression of the reference genes glyceraldehyde-3-phosphate dehydrogenase (*Gapdh*), β-actin (*Actb*), and β2-microglobulin (*B2m*). Means ± SEM of 3-5 experiments are shown. Statistical analysis compares the two osmotic conditions by unpaired *t*-test. (**C**) Surface expression of Na^+^,K^+^-ATPase in mIMCD3 cells exposed to norm- or hyperosmotic media for 72 h. Immunofluorescence microscopy was performed in non-permeabilized cells. Nuclei were counterstained with Hoechst 33342. Images are representative of three individual experiments. (**D**) Expression of α1-subunit of Na^+^,K^+^-ATPase in homogenate (Ho) and plasma membranes (PM) of mIMCD3 cells grown for 72 h in norm- or hyperosmotic media. Expression was determined by immunoblotting. β-actin was used as a loading control. Image represents three independent experiments. (PDF 361 kb)

Additional file 2: **Figure S2.** Verification of hLCN2-R isoform B expression in stable CHO-K1 cell transfectants. (**A**) Reverse transcription PCR of hLCN2-R-isoform B CHO-K1 clone D6.D10. RNA isolation and cDNA synthesis were carried out as described [31]. Primers and cycling conditions are listed in Table 1. (**B**) Immunoblot of pcDNA3.1 and hLCN2-R isoform B CHO-K1 cells. Cells were grown to confluency, washed once with PBS, harvested by scraping, and suspended in buffer containing 250mM sucrose, 5 mM Tris, pH7.5, and protease inhibitors. Homogenization by sonication and immunoblotting were carried out as described in the Methods. (PDF 96 kb)

Additional file 3: **Figure S3.** β-catenin siRNA reduces β-catenin mRNA expression in mIMCD3 cells exposed to norm- or hyperosmotic media. (**A**) mIMCD3 cells exposed to norm- or hyperosmotic media for 24 h were transfected with siRNA against β-catenin or control siRNA for 6 h, and expression levels of β-catenin mRNA were determined by PCR in norm- or hyperosmotic media after additional 24 h. (**B**) Silencing was stable between 16 and 48 h after transfection. *Gapdh* was used as reference gene. The experiments are representative of at least three similar ones. (PDF 199 kb)

Additional file 4: **Figure S4.** LPS induces Lcn2 expression and secretion and counteracts the effects of hyperosmolarity on Lcn2 and Lcn2-R expression in mIMCD3 cells. (**A**) Cells were cultured for 24 h in normosmotic medium with FBS, as described in the Methods and the medium was replaced by normosmotic medium without FBS ± LPS (5 μg/ml) for various time points. Medium was concentrated using Vivaspin 500 Centrifugal Concentrators (10 kDa MW cut-off) prior to immunoblotting. (**B**) Cells were cultured in normosmotic medium, as described in (**A**), prior to treatment with different concentrations of LPS for 18 h in the same medium without FBS. Medium was collected and Lcn2 secretion determined by immunoblotting, as described above. (**C**) mIMCD3 cells were cultured as described above and treated ± LPS (5 μg/ml) for 18 h in normosmotic medium without FBS prior to medium collection and measurement of Lcn2 secretion by immunoblotting. Cells were washed, scraped and homogenized by sonication in isosmotic sucrose buffer supplemented with protease inhibitors for immunoblotting. (**D**, **E**) mIMCD3 cells were exposed to norm- or hyperosmotic media for 24 h and treated ± LPS (5 mμg/ml) for additional 18 h in the same media without FBS prior to RNA isolation. RT-PCR shows mRNA expression for *Lcn2* (**D**), Lcn2-R (**E**) and the reference gene *Gapdh*. The experiments are representative of at least three similar ones. (PDF 96 kb)
