# Supplementary figures and images for: Tonicity inversely modulates lipocalin-2 (Lcn2/24p3/NGAL) receptor (SLC22A17) and Lcn2 expression via Wnt/β-catenin signaling in renal inner medullary collecting duct cells: implications for cell fate and bacterial infection
Source: Cell Commun Signal. 2018 Nov 7;16:74. doi: 10.1186/s12964-018-0285-3 (PMC6223074; doi:10.1186/s12964-018-0285-3)

**A**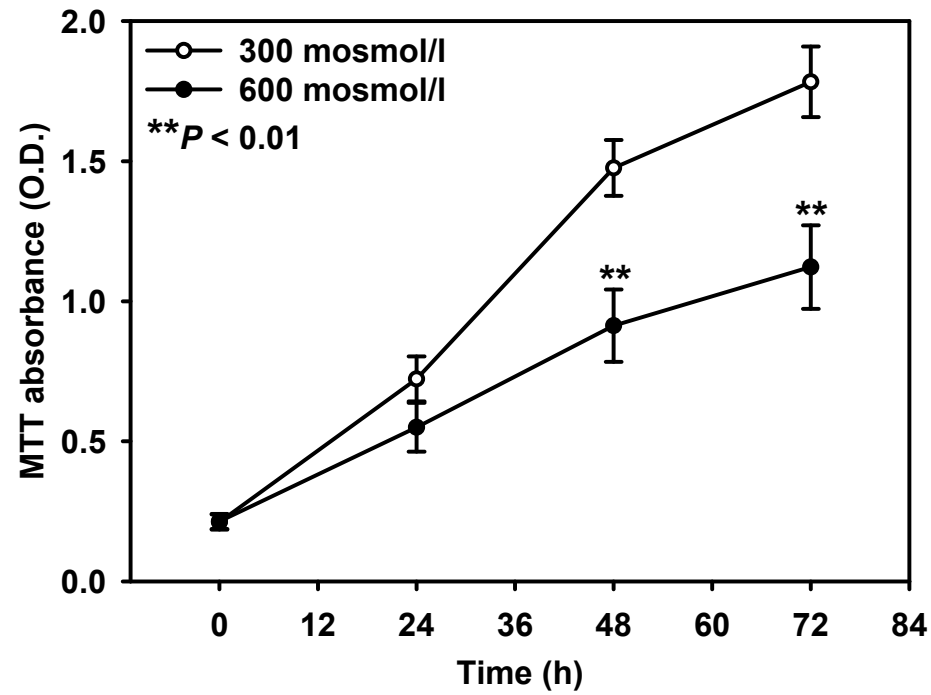**B**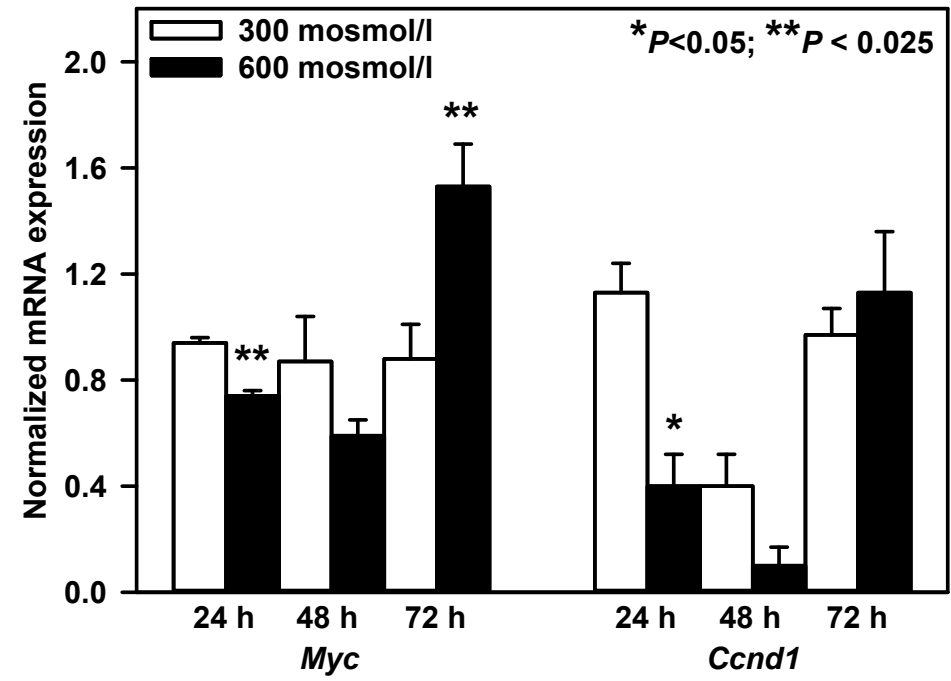**Suppl. Figure 1**

**C****300 mosmol/l**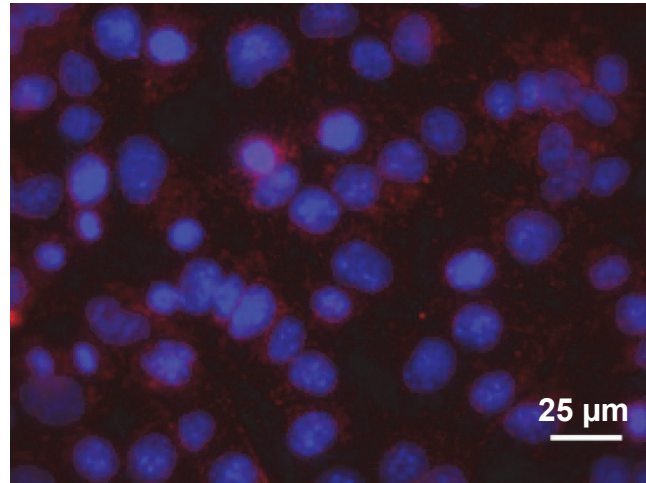**600 mosmol/l**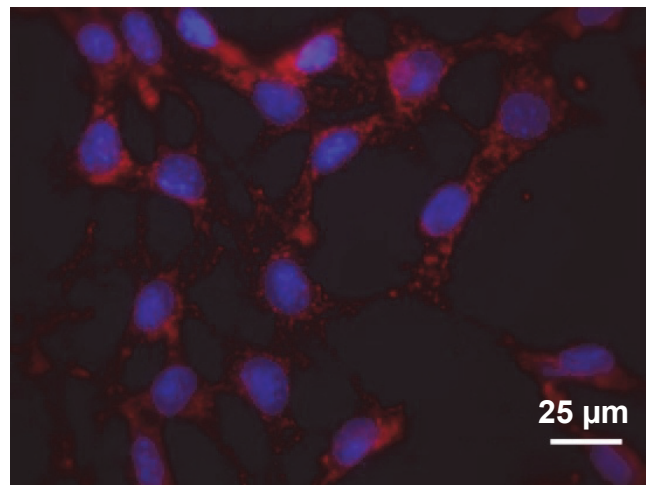**D**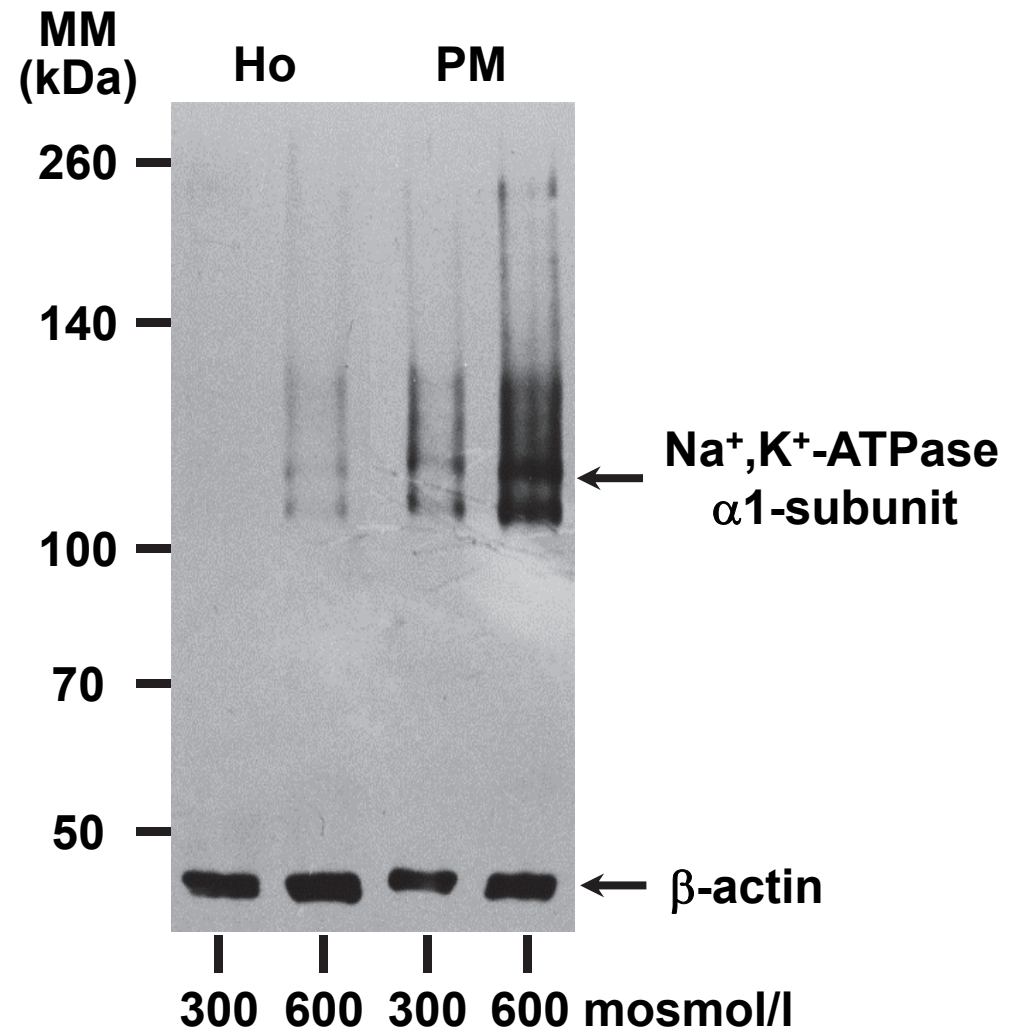**Suppl. Figure 1**

Supplement: Supplementary file 1 — Figure S1. Hyperosmolarity inhibits proliferation and induces adaptive responses in mIMCD3 cells. (A) Proliferation of mIMCD3 cells cultured in media without (normosmolarity) or with 100 mmol/l urea and NaCl (hyperosmolarity) for 72 h. Measurements of MTT absorbance/optical density (O.D.) reflecting cell proliferation were performed at indicated time points. Means ± SEM of 5 experiments are shown. Statistical analysis compares the two osmotic conditions at each time point by unpaired t-test. (B) Expression levels of proliferation genes c-Myc (Myc) and cyclin D1 (Ccnd1) by qPCR in mIMCD3 cells exposed to norm- or hyperosmotic media for 72 h. The data obtained were normalized to the expression of the reference genes glyceraldehyde-3-phosphate dehydrogenase (Gapdh), β-actin (Actb), and β2-microglobulin (B2m). Means ± SEM of 3-5 experiments are shown. Statistical analysis compares the two osmotic conditions by unpaired t-test. (C) Surface expression of Na+,K+-ATPase in mIMCD3 cells exposed to norm- or hyperosmotic media for 72 h. Immunofluorescence microscopy was performed in non-permeabilized cells. Nuclei were counterstained with Hoechst 33342. Images are representative of three individual experiments. (D) Expression of α1-subunit of Na+,K+-ATPase in homogenate (Ho) and plasma membranes (PM) of mIMCD3 cells grown for 72 h in norm- or hyperosmotic media. Expression was determined by immunoblotting. β-actin was used as a loading control. Image represents three independent experiments. (PDF 361 kb) [file 12964_2018_285_MOESM1_ESM.pdf]

**A**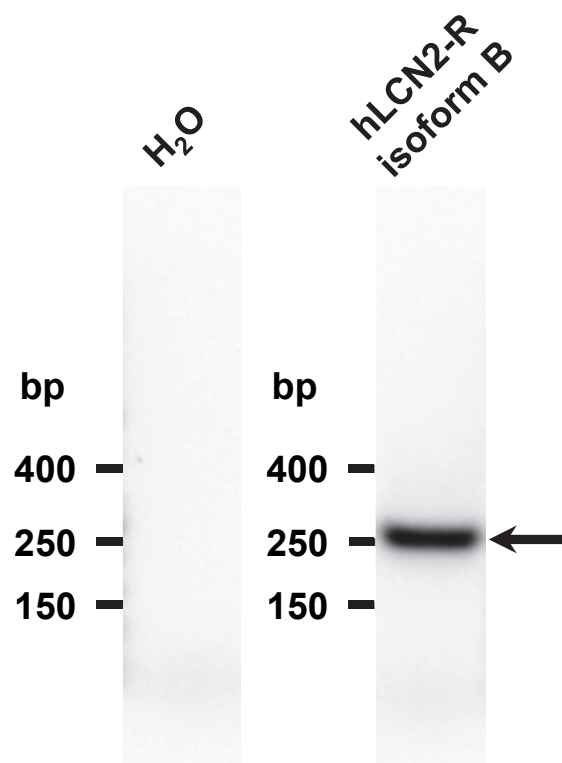**B**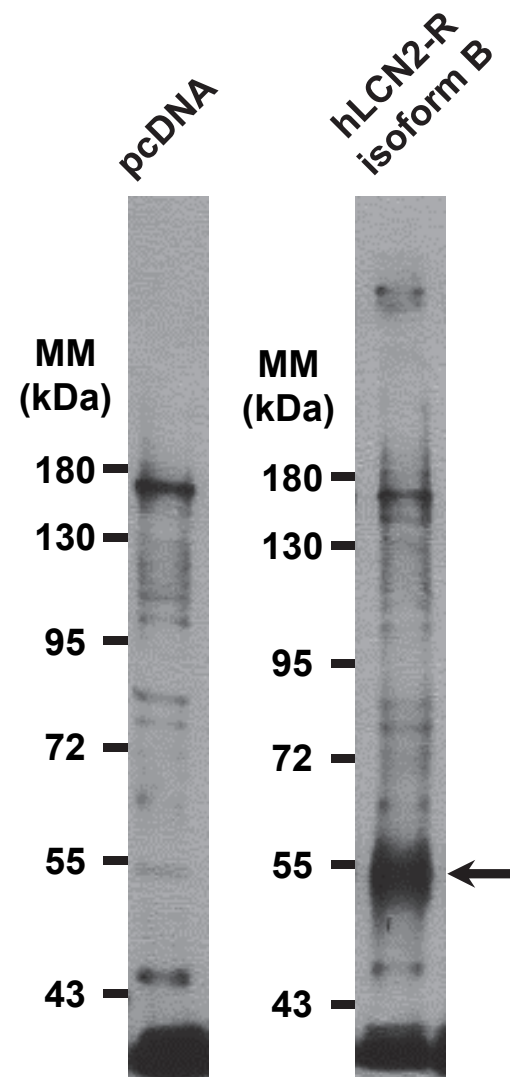

**Suppl. Figure 2**

Supplement: Supplementary file 2 — Figure S2. Verification of hLCN2-R isoform B expression in stable CHO-K1 cell transfectants. (A) Reverse transcription PCR of hLCN2-R-isoform B CHO-K1 clone D6.D10. RNA isolation and cDNA synthesis were carried out as described [31]. Primers and cycling conditions are listed in Table 1. (B) Immunoblot of pcDNA3.1 and hLCN2-R isoform B CHO-K1 cells. Cells were grown to confluency, washed once with PBS, harvested by scraping, and suspended in buffer containing 250mM sucrose, 5 mM Tris, pH7.5, and protease inhibitors. Homogenization by sonication and immunoblotting were carried out as described in the Methods. (PDF 96 kb) [file 12964_2018_285_MOESM2_ESM.pdf]

**A**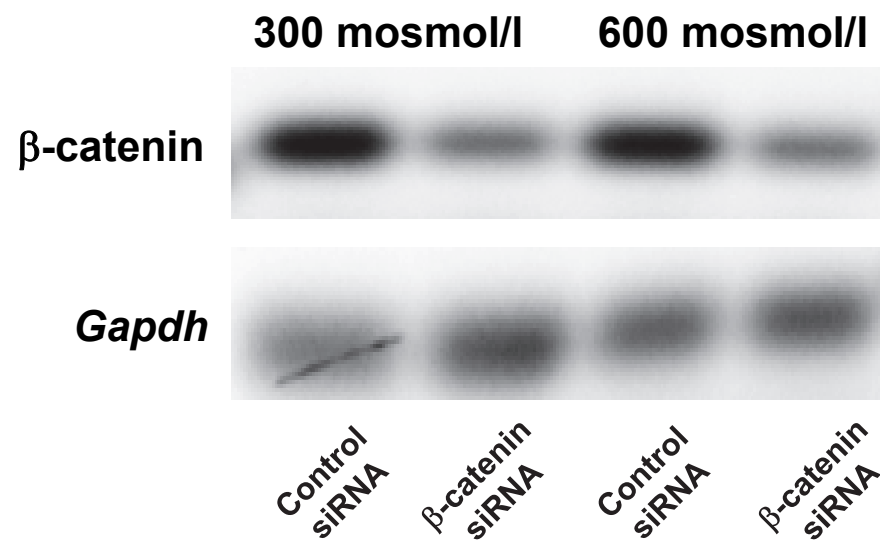**B**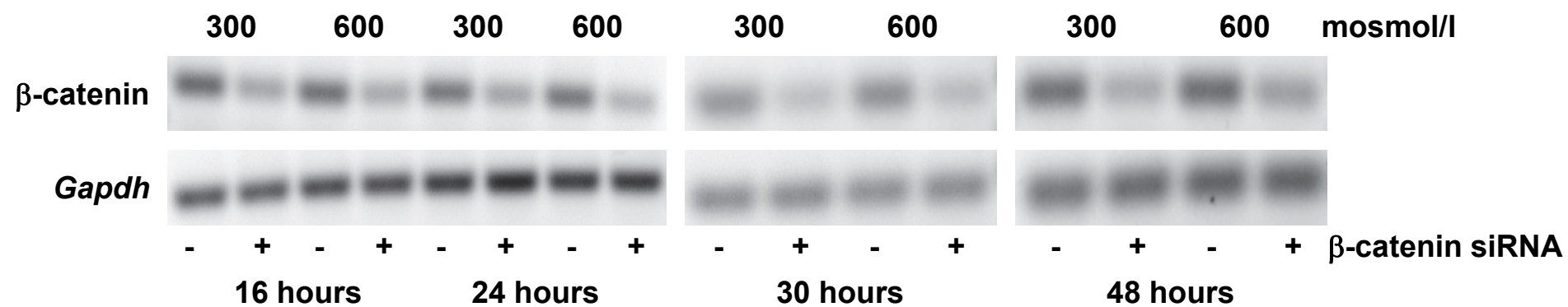

**Suppl. Figure 3**

Supplement: Supplementary file 3 — Figure S3. β-catenin siRNA reduces β-catenin mRNA expression in mIMCD3 cells exposed to norm- or hyperosmotic media. (A) mIMCD3 cells exposed to norm- or hyperosmotic media for 24 h were transfected with siRNA against β-catenin or control siRNA for 6 h, and expression levels of β-catenin mRNA were determined by PCR in norm- or hyperosmotic media after additional 24 h. (B) Silencing was stable between 16 and 48 h after transfection. Gapdh was used as reference gene. The experiments are representative of at least three similar ones. (PDF 199 kb) [file 12964_2018_285_MOESM3_ESM.pdf]

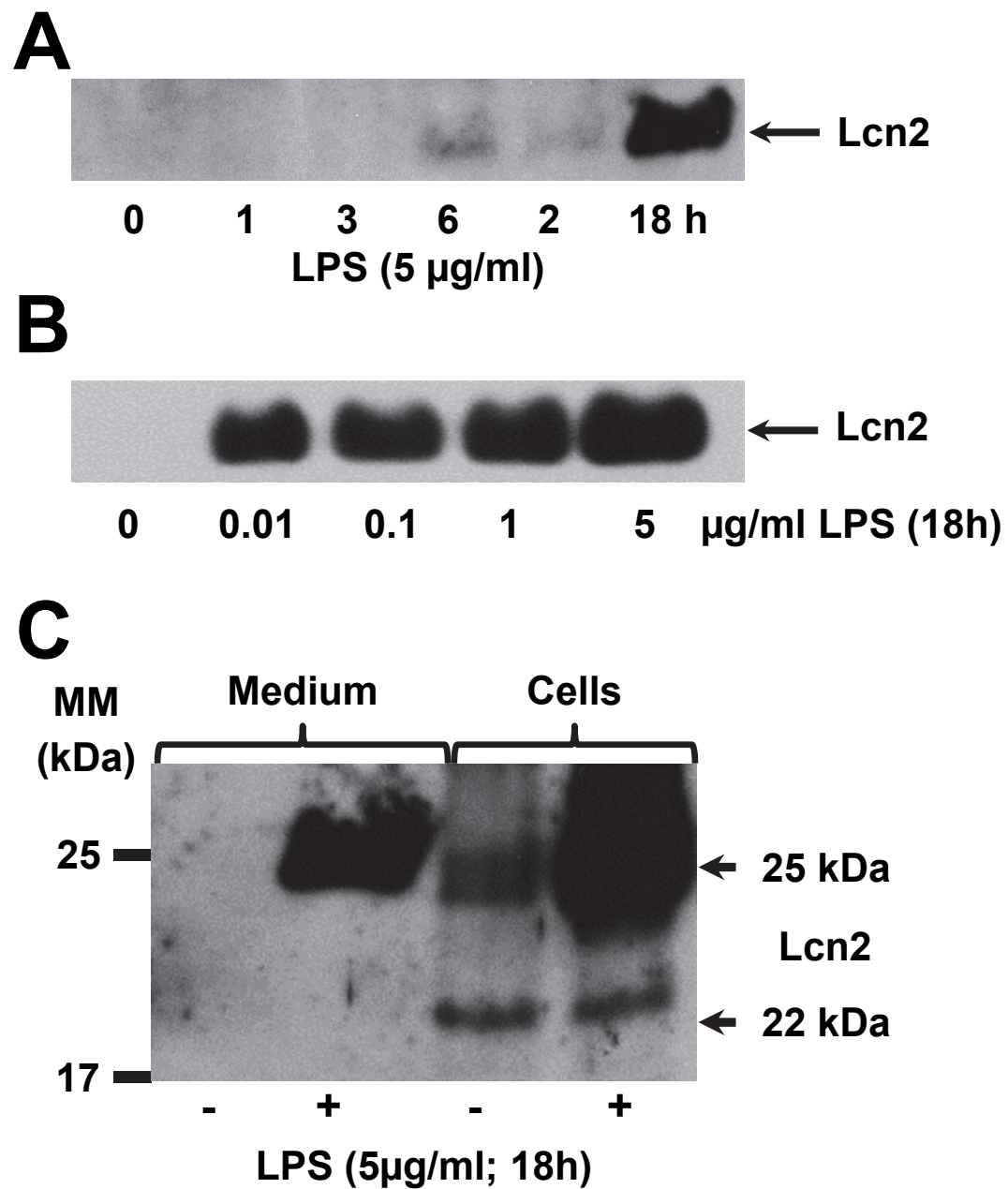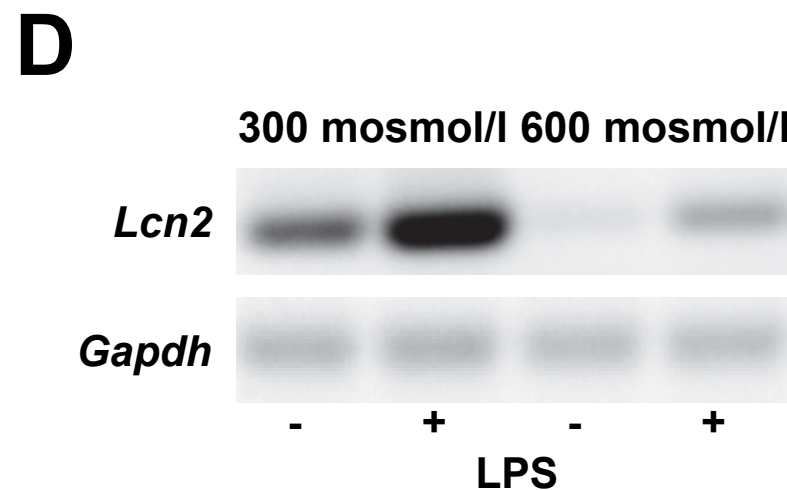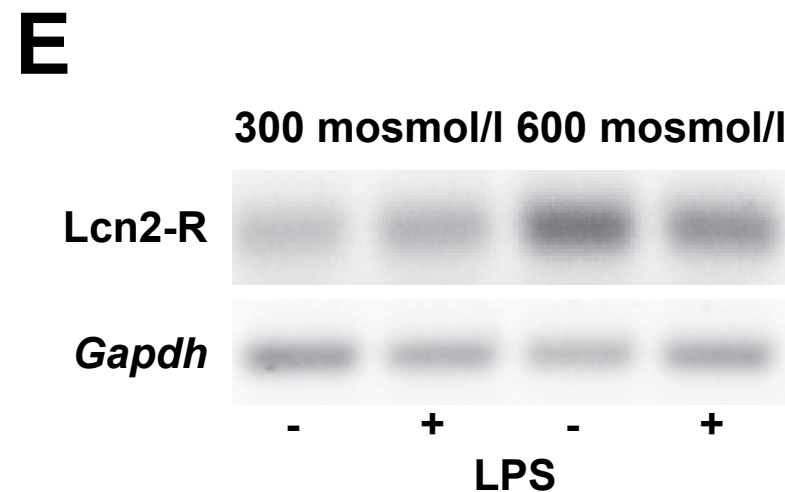

**Suppl. Figure 4**

Supplement: Supplementary file 4 — Figure S4. LPS induces Lcn2 expression and secretion and counteracts the effects of hyperosmolarity on Lcn2 and Lcn2-R expression in mIMCD3 cells. (A) Cells were cultured for 24 h in normosmotic medium with FBS, as described in the Methods and the medium was replaced by normosmotic medium without FBS ± LPS (5 μg/ml) for various time points. Medium was concentrated using Vivaspin 500 Centrifugal Concentrators (10 kDa MW cut-off) prior to immunoblotting. (B) Cells were cultured in normosmotic medium, as described in (A), prior to treatment with different concentrations of LPS for 18 h in the same medium without FBS. Medium was collected and Lcn2 secretion determined by immunoblotting, as described above. (C) mIMCD3 cells were cultured as described above and treated ± LPS (5 μg/ml) for 18 h in normosmotic medium without FBS prior to medium collection and measurement of Lcn2 secretion by immunoblotting. Cells were washed, scraped and homogenized by sonication in isosmotic sucrose buffer supplemented with protease inhibitors for immunoblotting. (D, E) mIMCD3 cells were exposed to norm- or hyperosmotic media for 24 h and treated ± LPS (5 mμg/ml) for additional 18 h in the same media without FBS prior to RNA isolation. RT-PCR shows mRNA expression for Lcn2 (D), Lcn2-R (E) and the reference gene Gapdh. The experiments are representative of at least three similar ones. (PDF 96 kb) [file 12964_2018_285_MOESM4_ESM.pdf]
